# Transcriptomic analysis comparing mouse strains with extreme total lung capacities identifies novel candidate genes for pulmonary function

**DOI:** 10.1186/s12931-017-0629-3

**Published:** 2017-08-09

**Authors:** Leema George, Ankita Mitra, Tania A. Thimraj, Martin Irmler, Sangeetha Vishweswaraiah, Lars Lunding, Dorothea Hühn, Alicia Madurga, Johannes Beckers, Heinz Fehrenbach, Swapna Upadhyay, Holger Schulz, George D. Leikauf, Koustav Ganguly

**Affiliations:** 1 SRM Research Institute, SRM University, Chennai, 603203 India; 2 Institute of Experimental Genetics, Helmholtz Zentrum Muenchen, German Research Center for Environmental Health, 85764 Neuherberg, Munich Germany; 3 Priority Area Asthma & Allergy, Division of Asthma Exacerbation & Regulation, Research Center Borstel, Airway Research Center North (ARCN), 23845 Borstel, Germany; 4 Department of Medicine, Pulmonary and Critical Care Medicine, University Medical Centre Giessen and Marburg, Philipps-University Marburg, Marburg, Germany; 5 Department of Internal Medicine (Pulmonology), University of Giessen and Marburg Lung Center (UGMLC), 35392, Giessen, Germany; 6 German Center for Diabetes Research (DZD), 85764 Neuherberg, Germany; 7 Experimental Genetics, Technische Universität München, 85354 Freising, Germany; 8 Priority Area Asthma & Allergy, Division of Experimental Pneumology, Research Center Borstel, Airway Research Center North (ARCN), 23845 Borstel, Germany; 9 Lung and Airway Research, Institute of Environmental Medicine, Karolinska Institutet, Box 287, SE-171 77 Stockholm, Sweden; 10 Institute of Lung Biology and Disease, Helmholtz Zentrum Muenchen, German Research Center for Environmental Health, 85764 Neuherberg, Munich Germany; 11 Institute of Epidemiology I, Helmholtz Zentrum Muenchen, German Research Center for Environmental Health, 85764 Neuherberg, Munich Germany; 12 Comprehensive Pneumology Center Munich (CPC-M), Munich, Germany; 13 Department of Environmental and Occupational Health, Graduate School of Public Health, University of Pittsburgh, Pittsburgh, PA 15219 USA; 14 Work Environment Toxicology; Institute of Environmental Medicine, Karolinska Institutet, Box 287, SE-171 77 Stockholm, Sweden; 15 Present address: Lahn-Dill-Kliniken, Klinikum Wetzlar, Medizinische Klinik II, Forsthausstraße 1, D-35578 Wetzlar, Germany

**Keywords:** Transcriptomics, Lung development, Chronic obstructive pulmonary disease, Asthma, WNT Signaling

## Abstract

**Background:**

Failure to attain peak lung function by early adulthood is a risk factor for chronic lung diseases. Previously, we reported that C3H/HeJ mice have about twice total lung capacity (TLC) compared to JF1/MsJ mice. We identified seven lung function quantitative trait loci (QTL: Lfnq1-Lfnq7) in backcross/intercross mice derived from these inbred strains. We further demonstrated, superoxide dismutase 3, extracellular (*Sod3*), *Kit* oncogene (*Kit*) and secreted phosphoprotein 1 (*Spp1*) located on these Lfnqs as lung function determinants. Emanating from the concept of early origin of lung disease, we sought to identify novel candidate genes for pulmonary function by investigating lung transcriptome in C3H/HeJ and JF1/MsJ mice at the completion of embryonic development, bulk alveolar formation and maturity.

**Methods:**

Design-based stereological analysis was performed to study lung structure in C3H/HeJ and JF1/MsJ mice. Microarray was used for lung transcriptomic analysis [embryonic day 18, postnatal days 28, 70]. Quantitative real time polymerase chain reaction (qRT-PCR), western blot and immunohistochemical analysis were used to confirm selected differences.

**Results:**

Stereological analysis revealed decreased alveolar number density, elastin to collagen ratio and increased mean alveolar volume in C3H/HeJ mice compared to JF1/MsJ. Gene ontology term “extracellular region” was enriched among the decreased JF1/MsJ transcripts. Candidate genes identified using the expression-QTL strategy include: ATP-binding cassette, sub-family G (WHITE), member 1 (*Abcg1),* formyl peptide receptor 1 (*Fpr1),* gamma-aminobutyric acid (GABA) B receptor, 1 (*Gabbr1)*; histocompatibility 2 genes: class II antigen E beta (*H2-Eb1*), D region locus 1 (*H2-D1*), and Q region locus 4 (*H2-Q4*); leucine rich repeat containing 6 (testis) *(Lrrc6),* radial spoke head 1 homolog (*Rsph1),* and surfactant associated 2 (*Sfta2).* Noteworthy genes selected as candidates for their consistent expression include: Wnt inhibitor factor 1 (*Wif1*)*,* follistatin *(Fst),* chitinase-like 1 (*Chil1*), and *Chil3*.

**Conclusions:**

Comparison of late embryonic, adolescent and adult lung transcript profiles between mouse strains with extreme TLCs lead to the identification of candidate genes for pulmonary function that has not been reported earlier. Further mechanistic investigations are warranted to elucidate their mode of action in determining lung function.

**Electronic supplementary material:**

The online version of this article (doi:10.1186/s12931-017-0629-3) contains supplementary material, which is available to authorized users.

## Background

The origin of many chronic lung diseases may be rooted early in life because of failure to achieve optimal peak lung function by early adulthood [1–3]. Lung dysfunction may develop in utero and during early life suggesting an underlying impairment of lung development, growth and maturation [4]. For example, persistent wheezing in the early years and lower lung function by age 6 years among children is associated with later onset of asthma. Similarly, following a cohort over 22 years, Lange et al. [5] reported that low forced expiratory volume in 1 s (FEV_1_) before age 40 as a risk factor for chronic obstructive pulmonary disease (COPD). Diminished lung function and hindered lung development is likely due to multiple environmental (e.g., maternal smoking) and genetic factors [6–18]. Thus, accumulating evidence suggests that early life determinants of lung function are possible determinants of obstructive airways diseases later in life. Therefore, in this study we sought to identify genetic determinants of lung function development that could provide important insight into the predisposition of chronic lung disease.

Evolving from the mouse phenome project, we previously assessed lung function in several inbred mouse strains [19–21]. The greatest differences were noted in C3H/HeJ as compared to JF1/MsJ mice. In male C3H/HeJ mice, total lung capacity (TLC) and specific TLC (TLC/body weight) = 1837 μl and 64 μl/g, respectively. In male JF1/MsJ mice, TLC = 904 μl and specific TLC = 40 μl/g. A similar difference was observed in female mice (C3H/HeJ: TLC = 1443 μl and specific TLC 65 μl/g and JF1/MsJ: TLC = 874 μl and specific TLC 53 μl/g). Contrasting C3H/HeJ and JF1/MsJ back- and inter-cross mice, lung function phenotyping was followed by quantitative trait loci (QTL) analysis identifying lung function QTLs (Lfnq1 – Lfnq7) [20]. Consistent with the early life impairment concept, JF1/MsJ mice were more susceptible to lung injury following exposure to carbon nanoparticle (CNP) and acrolein (a toxic component of tobacco smoke) compared to C3H/HeJ [22–24].

Examining the mouse lung function QTL regions Lfnq1 and Lfnq2 located on mouse chromosome (mCh) 5 [20], we previously demonstrated that superoxide dismutase 3 extracellular (*Sod3*), Kit oncogene (*Kit*) and secreted phosphoprotein 1 (*Spp1*) as determinants of lung function in mice [21, 25–27]. *SOD3* variants were also associated with declined lung function in children [25]. When compared to strain-matched C57BL/6 J control mice, gene-targeted *Sod3* ^*(−/−)*^ mice exhibited poor ventilation efficiency [21]; *Kit* mutant mice (*Kit* ^*W-sh*^ */* ^*W-sh*^) exhibited increased TLC, residual volume, lung compliance (C_L_), and alveolar size [27]; and gene-targeted *Spp1* ^*(−/−)*^ mice exhibited decreased TLC and increased specific C_L_ and alveolar size [26].

In this study, we examined differences in lung architecture and lung transcripts in these highly divergent mouse strains. Transcriptomic analysis was performed at three strategic times covering completion of embryonic lung development, bulk alveolar formation and growth. Morphometric studies in the adult mouse lung were performed to identify any structural alterations arising from developmental differences between C3H/HeJ and JF1/MsJ mice. Differential transcript expression was also used to identify genes associated with TLC QTLs located within Lfnq3 on mCh15 (40.3–72.5 Mbp) and Lfnq4 on mCh17 (10.9–39.0 Mbp) associated with TLC located within Lfnq3 on mCh15 (40–73 Mbp) and Lfnq4 on mCh17 (10–39 Mbp).

## Methods

### Mice

All procedures were approved by the Bavarian Animal Research Authority, Animal Research Authority of Schleswig-Holstein, and University of Pittsburgh, PA. Frozen and paraffin embedded tissues were procured to carry out experiments at SRM University, India according to the Institutional Animal Ethics Committee (IAEC) permission. Mouse strains C3H/HeJ (#000659) and JF1/MsJ (#003720) were purchased from Jackson laboratory (Bar Harbor, ME, USA) and housed in specific pathogen free conditions. Food and water were available ad libitum. Three developmental stages, namely, embryonic day (E) 18 (completion of embryonic lung development), postnatal day (P) 28 (completion of bulk alveolar formation) and P70 (completion of lung growth and maturity) were selected for the microarray studies. E18 embryos (days post coitum) were stage matched. Mice were anesthetized (4 mg/kg xylene and 188 mg/kg ketamine i.p.) and the posterior aorta was severed. To obtain tissue for mRNA and protein analysis (*n* = 5 mice/strain/stage, female), the diaphragm was punctured, and the chest cavity opened. Lungs were excised, frozen in liquid nitrogen, and stored (−80 °C). Whole lung was used for RNA extraction in case of stage E18. The left lobe of the lung was used for RNA extraction and the right lobe was used for protein extraction for P28 and P70.

### Design-based stereological analysis of lung structures and lung function measurements

Comparison of age and sex matched adult C3H/HeJ and JF1/MsJ mice was performed according to American Thoracic Society/European Respiratory Society guidelines [28]. Use of a cascade design allowed linking data retrieved at the light microscopic level using a PC-based software tool (newCAST, Visiopharm, Hersholm, Denmark) with the data obtained at the transmission electron microscope (TEM) [29]. Alveolar numbers were quantified as described previously [30]. Lungs were fixed (20 cm H_2_0) in situ by intra-tracheal instillation of a mixture of 1% glutaraldehyde (Serva, Germany), 1% paraformaldehyde in 0.1 M sodium cacodylate solution (Merck, Germany) [31]. After 20 min, the trachea was ligated and the lungs were fixed overnight (4 °C). Lung volume was determined by fluid displacement. To obtain systematic uniform random (SUR) samples, lungs were embedded (2% agar-agar) and sectioned (2 mm). Tissues were randomly embedded into glycolmethacrylate (GMA, Technovit 7100, HeraeusKulzer, Germany) for light microscopy or epoxy resin (Araldite, Serva, Germany) for transmission electron microscopy. For light microscopy, samples were stained with 1% OsO_4_ in 0.1 M sodium cacodylate solution (Merck, Germany), followed by half-saturated aqueous uranyl acetate (Agar Scientific, UK), acetone dehydrated GMA embedded, and sectioned (2 μm). For TEM analysis, SUR sampling of tissue blocks (1x1x1 mm^3^) was performed. Tissue blocks were post-fixed with 1% OsO_4_ in 0.1 M sodium cacodylate solution followed by 3% aqueous tannic acid (Mallinckrodt, USA) staining for elastin [32].

### Lung function measurements

Lung functions were measured twice with air or twice with saline filled lung as described previously [33]. Saline enables exclusion of surface tension. Lungs were filled to TLC (30 cm H_2_O), emptied in 0.1 ml steps, and volume and corresponding plateau pressures were determined after each step. C_L_ was determined as the linear slope of the pressure-volume curve.

### RNA and Protein extraction

Lung RNA was isolated (RNeasy #74104; Qiagen, Hilden, Germany), quantified (Thermofisher nanodrop 2100), and quality assessed (Agilent Bioanalyzer). For protein analysis, lung was homogenized using 50 mM TrisHCl, 150 nM NaCl, NP-40-1%; sodium desoxycholate – 0.5%*W*/*V*; SDS-0.1%, radio immunoprecipitation solution (RIPA buffer #RB4476 Biobasic; Ontario, Canada) in 1:100 ratio supplemented with protease inhibitors (# ab6562 Abcam, Cambridge, UK) and centrifuged (10,000×g, 15 min, 4 °C). The supernatant was aspirated. Total protein content was determined spectrophotometrically (620 nm Protein Assay Dye Reagent # 500–0006; BioRad, USA).

### Transcript and protein analyses

Transcriptome-wide analysis of steady state mRNA levels was analyzed by microarray (*n* = 5/strain/stage, GeneChip®MouseExon 1.0 ST Array Cat. #902473, Affymetrix, Santa Clara, CA). Selected candidates were confirmed by quantitative real time - polymerase chain reaction (qRT-PCR) and western blot as described previously [21, 25]. Total RNA was amplified (GeneChip®Whole Transcript (WT) Sense Target Labeling Assay) without rRNA depletion. Expression console (v.1.3.0.187, Affymetrix) was used for quality control and to obtain annotated normalized RMA data (setting: Gene Level – Extended: RMA-Sketch). The dataset was filtered for probesets with an annotation for gene symbols and only the probeset with the highest number of probes per gene was used in statistical analysis. Array data has been submitted to the Genome Expression Omnibus (GEO) database at National Center for Biotechnology Information NCBI (GSE80078). For qRT-PCR, Quantitech primers (Qiagen) for Wnt inhibitory factor 1 (WIF1 Cat. # QT01065848), frizzled homolog 6 (FZD6 Cat. # QT00109998), and beta actin (ACTB Cat. # QT00095242) were used. ACTB was used as the reference control. For western blot analysis primary antibody for chitinase-like 3 (CHI3L3, Cat. # ab192029, Abcam) and horse radish peroxidase conjugated goat anti-rabbit antibody (Cat. # ab97051, Abcam) was used as the secondary antibody. Clarity Western ECL substrate (Cat. # 170–50,605; Biorad) was used for immunodetection (Syngene G: Box). The blots were stripped using Restore western blot stripping buffer (Cat # 21059, Thermofisher, Waltham, MA) for reprobing with ACTB primary antibody (Cat. # ab8227, Abcam).

### Immunohistochemistry

Lungs were fixed (20 cm H_2_O) in situ with 4% phosphate buffered formaldehyde, embedded into agar-agar blocks, and sectioned (2 μm) for SUR sampling. Deparaffinized sections were subjected to antigen retrieval by microwaving slides (3 X 5 min, 20% power) in 1 mM citrate solution (pH 6.0). Slides were rehydrated in phosphate-buffered saline, followed by immersion into a mixture of methanol and H_2_O_2_ (Cat. # 1.08597.1000; Merck, Darmstadt, Germany) (30 min) to inhibit endogenous peroxidases. Tissues were treated with 5% horse serum or 4% bovine serum albumin (BSA) in PBS (45 min, 20 °C) or goat serum in 1:10 ratio (20 min). Primary antibody omission was used as a negative staining control. Mouse WIF1 rabbit polyclonal primary antibody (Cat. #ab186845, Abcam), mouse FZD6 polyclonal goat antibody (Cat. # AF1526; R&D Systems, McKinley, MN), or follistatin (FST) polyclonal goat antibody (Cat. # AF669; R&D Systems) were used. Biotinylated horse anti-goat antibody (Cat. # BA9500; Vector Laboratories, Burlingame, CA) was used as secondary antibody. Vectastain elite ABC kit (Cat. # PK-6100; Vector Laboratories) was used with diaminobenzidine (DAB) reagent (Merck KGaA). Tissues were counterstained with Meyer’s hematoxylin (Cat. # 1.09249.1000; Merck KGaA).

### Statistics

Two-way ANOVA followed by all pairwise multiple-comparison procedures (SigmaStat 11.0 software; Systat Software Incorporation) were used to compare group means. *P* < 0.05 was considered as significant in all cases. For microarray data, statistical analyses were performed by utilizing the statistical programming environment R [34] implemented in CARMAweb [35]. Genewise testing for differential expression used *t*-test and Benjamini-Hochberg multiple testing correction [false discovery rate (FDR) ≤ 0.10]. Gene ontology (GO) term and pathway enrichment analyses were done with Database for Annotation, Visualization and Integrated Discovery (DAVID) v6.7 (*p* < 0.05, FDR ≤ 0.05) [36].

## Results

### Design-based stereological analysis of lung architecture

Previously, we noted that mice C3H/HeJ mice had increased TLC and TLC/Bw than age and sex-matched JF1/MsJ mice [20]. To assess these differences further, we employed design-based stereological analysis to examine the lung architecture of each strain. The light and TEM comparison of age- and sex-matched lungs of JF1/MsJ and C3H/HeJ mice revealed significantly different alveolar architecture at an age of 12–14 weeks (Table 1, Additional file 1: Figure S1). Interestingly, C3H/HeJ mice had increased mean chord length and mean alveolar volume but decreased alveolar number density and elastin to collagen ratio compared to JF1/MsJ mice. These structural differences are consistent with the functional measures of increased C_Lair_, C_Lsaline,_ and specific C_Lsaline_ and ratio of C_L-saline_/C_L-air_ in C3H/HeJ compared to JF1/MsJ mice. Increased compliance could result from decreased elasticity and surface tension of the larger alveoli of C3H/HeJ lung. Because alveolar number and size were complementary, the lungs of both mouse strains exhibit near equal total alveolar surface area. To achieve higher alveolar surface area for adequate gas exchange, the smaller lung volume of JF1/MsJ mice would require more alveolar septa.

**Table 1:** Morphometric evaluation of C3H/HeJ and JF1/MsJ mouse lung

Morphometric Parameters	C3H/HeJ	JF1/MsJ	*P* value	C3H/HeJ	JF1/MsJ	*p* value
Number of mice	MALE: *n* = 5			FEMALE: *n* = 3–5		
Age (weeks)	13	12–14		13	12–14	
Fixed lung volume (μl)	1092 ± 123	735 ± 71	0.001	987 ± 54	667 ± 23	0.001
Mean chord length (Lcm) (μm)	74.3 ± 9.9	48.5 ± 5.5	0.002	69.2 ± 14.8	47.3 ± 2.0	0.011
Mean alveolar volume (10^3^/μm^3^)	61.1 ± 7.9	40.8 ± 5.9	0.01	71.5 ± 11.4	42.3 ± 7.0	0.007
Alveolar number density (10^6^/cm^3^ parenchyma)	10.2 ± 1.1	14.7 ± 2.0	0.005	8.8 ± 1.5	14.5 ± 2.2	0.001
Alveolar septum width (μm)	0.72 ± 0.07	0.66 ± 0.08	ns	0.82 ± 0.14	0.63 ± 0.11	0.005
Total number of alveoli (10^6^/both lungs)	9.2 ± 1.1	8.9 ± 1.7	ns	6.9 ± 1.4	8.2 ± 1.6	ns
Total alveolar surface area (cm^2^)	952 ± 88	957 ± 125	ns	847 ± 70	906 ± 102	ns
Elastin - collagen ratio	1.40 ± 0.3	1.95 ± 0.3	0.004	1.83 ± 0.3	2.12 ± 0.2	0.035
Absolute volumes						
Parenchymal volume (mm^3^)	933 ± 112	629 ± 72	0.001	849 ± 71	571 ± 28	0.001
Non-parenchymal volume (mm^3^)	145 ± 33	98 ± 23	0.002	126 ± 22	78 ± 11	0.003
Alveolar airspace volume (mm^3^)	574 ± 73	377 ± 53	0.001	514 ± 41	340 ± 21	0.001
Alveolar duct airspace volume (mm^3^)	261 ± 32	167 ± 26	0.041	237 ± 28	155 ± 15	ns
Alveolar septal volume (mm^3^)	69 ± 12	63 ± 5	ns	69 ± 11	58 ± 5	ns
Total alveolar surface density (μm^2^/μm^3^)	0.10 ± 0.01	0.15 ± 0.01	ns	0.11 ± 0.01	0.16 ± 0.01	ns
Alveolar epithelial type I cells (mm^3^)	19.5 ± 3.3	19.2 ± 3.2	ns	20,8 ± 4.02	15.5 ± 3.45	ns
Alveolar epithelial type II cells (mm^3^)	6.0 ± 2.8	7.8 ± 4.6	ns	4.3 ± 1.5	6.5 ± 2.7	ns
Capillary endothelial cells (mm^3^)	16.3 ± 3.7	13.6 ± 1.4	ns	15.8 ± 1.9	11.8 ± 2.5	ns
Interstitial tissue (mm^3^)	22.7 ± 5.3	17.5 ± 2.1	ns	22.5 ± 4.5	19.3 ± 6.1	ns
Total alveolar surface area (cm^2^)	952 ± 88	957 ± 125	ns	847 ± 70	906 ± 102	ns
Volume densities						
Alveolar epithelial type I cells (mm^3^/mm^3^)	28.2 ± 1.5	30.5 ± 4.2	ns	30.2 ± 2.1	27.23 ± 2.4	ns
Alveolar epithelial type II cells (mm^3^/mm^3^)	9.0 ± 4.2	12.5 ± 6.7	ns	6.3 ± 3.4	10.9 ± 4.0	ns
Capillary endothelial cells (mm^3^/mm^3^)	23.3 ± 2.3	21.7 ± 3.1	ns	22.8 ± 1.9	21.2 ± 2.5	ns
Interstitial tissue (mm^3^/mm^3^)	32.9 ± 3.7	28.2 ± 2.6	ns	32.5 ± 3.0	33.2 ± 3.1	ns
Collagen (mm^3^/mm^3^)	2.78 ± 0.49	2.63 ± 0.51	ns	3.02 ± 0.62	2.42 ± 0.41	ns
Elastin (mm^3^/mm^3^)	3.87 ± 1.23	5.08 ± 1.06	ns	5.3 ± 1.55	5.08 ± 0.72	ns
Lung function	*n* = 4	*n* = 3		n-3	n-3	
Total Lung capacity (TLC) (μl)	1425 ± 164	750 ± 50	<0.05	1467 ± 125	800 ± 82	<0.05
Compliance _air_ (μl/cm H_2_0)	143 ± 5	36 ± 7	<0.05	130 ± 6	34 ± 6	<0.05
Compliance _saline_ (μl/cm H_2_0)	306 ± 46	96 ± 13	<0.05	296 ± 37	107 ± 20	<0.05
Specific compliance _saline_ (μl/cm H_2_0)	214 ± 15	128 ± 5	<0.05	202 ± 9	134 ± 23	<0.05
Compliance _saline_/Compliance _air_	2.2 ± 0.4	2.7 ± 0.1	<0.05	2.3 ± 0.2	3.2 ± 0.9	<0.05

*ns* not significant

Values are mean ± S.D. Significant difference between sex-matched mouse strains was determined by ANOVA

### Transcript analysis in the lung (E18, P28 and P70)

The number of increased and decreased transcripts (fold change ≥ |2|-fold and FDR < 0.10) in JF1/MsJ mice compared to C3H/HeJ was: (i) E18: 45 increased and 102 decreased; (ii) P28: 65 increased and 109 decreased; and (iii) P70: 46 increased and 127 decreased (Additional file 1: Table S1-S6). Using DAVID, decreased transcripts were found to be enriched in the gene ontology category “Extracellular region” (GO: 00005576) (Table 2) at E18 and P70. This was consistent with the observed strain specific differences in elastin to collagen ratio and lung architecture. Collagen type XXVIII, alpha 1 (COL28A1) transcript was decreased by −2.8 fold in JF1/MsJ lungs compared to C3H/HeJ at E18 and P70. At E18, P28, and P70, 12 transcripts increased and 22 decreased in JF1/MsJ compared to C3H/HeJ (Table 3). At stages E18 and P28, 16 transcripts increased and 28 decreased in JF1/MsJ compared to C3H/HeJ (Additional file 1: Table S7). At stages P28 and P70, 32 transcripts increased and 70 decreased lung transcripts in JF1/MsJ compared to C3H/HeJ (Additional file 1: Table S8).

**Table 2:** Extracellular region is an enriched gene ontology category (GO: 0005576) associated with the significantly decreased transcripts in JF1/MsJ vs C3H/HeJ mouse lung at embryonic day 18 (E18) and postnatal day 70 (P70)

Gene	Gene symbol	Entrez ID	Fold Difference
E18 decreased transcripts of GO term extracellular region; Enrichment Score = 2.83; *p* = 0.0005			
Chemokine (C-X-C motif) ligand 15	*Cxcl15*	20,309	−14.6
Hemolytic complement	*Hc*	15,139	−8.61
Kallikrein 1-related peptidase b21	*Klk1b21*	16,616	−8.25
Surfactant associated protein A1	*Sftpa1*	20,387	−3.80
Collagen, type XXVIII, alpha 1	*Col28a1*	213,945	−3.15
Paraoxonase 3	*Pon3*	269,823	−2.96
Ceruloplasmin	*Cp*	12,870	−2.95
Neuron-derived neurotrophic factor	*Ndnf*	68,169	−2.41
Fibroblast growth factor 1	*Fgf1*	14,164	−2.21
EGF-like, fibronectin type III and laminin G domains	*Egflam*	268,780	−2.13
Transferrin	*Trf*	22,041	−2.12
Interleukin 33	*Il33*	77,125	−2.04
P70 decreased transcripts of GO term extracellular region; Enrichment Score = 4.18; *p* = 0.04			
Hemolytic complement	*Hc*	15,139	−27.88
Chitinase 3-like 3	*Chi3l3*	12,655	−15.93
Kallikrein 1-related peptidase b21	*Klk1b21*	16,616	−11.92
Hepcidin antimicrobial peptide	*Hamp*	84,506	−4.87
Chemokine (C-C motif) ligand 6	*Ccl6*	20,305	−3.92
Matrix metallopeptidase 3	*Mmp3*	17,392	−3.62
Insulin-like growth factor binding protein 6	*Igfbp6*	16,012	−2.83
Collagen, type XXVIII, alpha 1	*Col28a1*	213,945	−2.76
Angiopoietin-like 7	*Angptl7*	654,812	−2.72
Leptin receptor	*Lepr*	16,847	−2.28
Chitinase, acidic	*Chia*	81,600	−2.26
Oxidized low density lipoprotein (lectin-like) receptor 1	*Olr1*	108,078	−2.21
Coagulation factor VII	*F7*	14,068	−2.18
Paraoxonase 3	*Pon3*	269,823	−2.18
Plasminogen activator, tissue	*Plat*	18,791	−2.10
Secreted Ly6/Plaur domain containing 1	*Slurp1*	57,277	−2.02

Values are mean fold difference of (JF1/MsJ)/(C3H/HeJ) for lung transcript levels (cut off for fold change ≥2 fold; false discovery rate 0.10)

**Table 3:** Lung transcripts (34 genes) consistently increased (12) or decreased (22) across embryonic stage 18 (E18), postnatal stage (P) 28 and P70 in JF1/MsJ (JF1) mice compared to C3H/HeJ (C3H)

Gene Name	Gene symbol	Entrez ID	Probeset	Fold Difference		
				E18	P28	P70
Glutaredoxin 3	*Glrx3*	30,926	6,923,313	12.22	6.08	3.27
RIKEN cDNA G730007D18 gene	*G730007D18Rik*	100,038,502	7,006,004	3.66	2.87	2.41
Holliday junction recognition protein	*Hjurp*	381,280	6,760,490	3.58	2.09	2.20
Synaptonemal complex central element protein 1	*Syce1*	74,075	6,972,143	3.00	2.06	2.13
Predicted gene 14,403	*Gm14403*	433,520	6,883,977	2.98	2.79	2.68
RIKEN cDNA 2,610,028 J07 gene	*2610028J07Rik*	71,813	6,859,453	2.94	2.74	2.82
Microspherule protein 1	*Mcrs1*	51,812	6,918,337	2.62	2.03	2.30
RIKEN cDNA 2610507I01 gene	*2610507I01Rik*	72,203	6,788,659	2.60	5.35	3.74
Olfactory receptor 1061	*Olfr1061*	259,022	6,888,451	2.51	2.81	3.77
Olfactory receptor 170	*Olfr170*	258,959	6,844,403	2.47	4.67	3.43
Uncharacterized protein C130090J04	*C130090J04*	328,049	6,860,959	2.36	2.12	2.20
RIKEN cDNA 6,330,403 K07 gene	*6330403K07Rik*	103,712	6,789,483	2.32	7.15	6.56
Glucagon-like peptide 1 receptor	*Glp1r*	14,652	6,849,762	−2.02	−2.33	−2.06
Solute carrier family 39 (metal ion transporter), member 8	*Slc39a8*	67,547	6,901,634	−2.07	−2.43	−2.51
Sodium channel, voltage-gated, type III, alpha	*Scn3a*	20,269	6,887,324	−2.08	−5.31	−4.49
Sterile alpha motif domain containing 9-like	*Samd9l*	209,086	6,951,281	−2.08	−2.74	−3.05
Interferon-induced protein 44	*Ifi44*	99,899	6,910,592	−2.10	−2.86	−2.19
Transmembrane protein 181A	*Tmem181a*	77,106	6,848,520	−2.13	−2.60	−2.40
NME/NM23 family member 7	*Nme7*	171,567	6,754,701	−2.13	−2.49	−2.70
Protein phosphatase 1, regulatory (inhibitor) subunit 3A	*Ppp1r3a*	140,491	6,951,783	−2.19	−2.10	−3.06
Megalencephalic leukoencephalopathy with subcortical cysts 1 homolog (human)	*Mlc1*	170,790	6,837,773	−2.24	−2.78	−3.13
Cytochrome P450, family 2, subfamily J, polypeptide 6	*Cyp2j6*	13,110	6,923,520	−2.29	−2.27	−2.39
RIKEN cDNA A330076H08 gene	*A330076H08Rik*	320,026	6,967,799	−2.35	−2.53	−2.06
Aminolevulinate, delta-, dehydratase	*Alad*	17,025	6,922,241	−2.82	−2.54	−2.67
RIKEN cDNA D230046O15 gene	*D230046O15Rik*	106,824	6,856,766	−3.00	−3.63	−2.54
Nuclear paraspeckle assembly transcript 1 (non-protein coding)	*Neat1*	66,961	6,867,774	−3.54	−3.45	−2.70
Surfactant associated 2	*Sfta2*	433,102	6,850,183	−4.02	−2.19	−2.23
Transmembrane protein 181A	*Tmem181a*	77,106	6,812,918	−4.26	−4.08	−3.40
RIKEN cDNA B930063I24 gene	*B930063I24Rik*	319,330	6,756,745	−5.06	−4.33	−6.19
G protein-coupled receptor 137B	*Gpr137b*	83,924	7,003,081	−5.39	−5.38	−3.06
Zinc finger, X-linked, duplicated B	*Zxdb*	668,166	7,021,109	−6.18	−7.68	−7.23
Kallikrein 1-related peptidase B21	*Klk1b21*	16,616	6,960,239	−8.25	−19.87	−11.92
Hemolytic complement	*Hc*	15,139	6,886,022	−8.61	−32.16	−27.88
RIKEN cDNA A730017L22 gene	*A730017L22Rik*	613,258	6,890,967	−13.13	−15.62	−14.60

Values are mean fold change for transcripts (cut off for fold change ≥2 fold; false discovery rate = 0.10

### Evaluation of selected transcripts

**Fig. 1:**
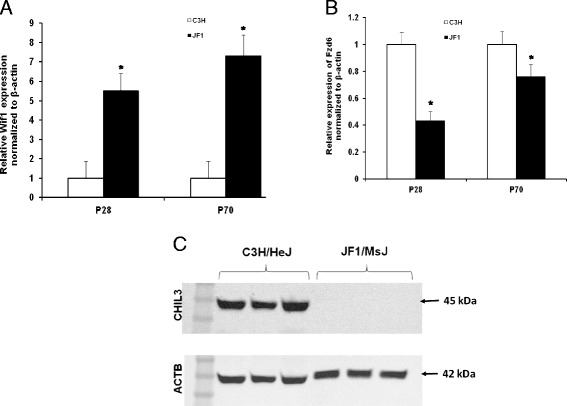
Comparative expression of lung Wnt inhibitor factor 1 (*Wif1*) and frizzled homolog 6 (*Fzd6*) and chitinase 3 like 3 (CHIL3) in JF1/MsJ and C3H/HeJ mice. **a** Expression of lung *Wif1* mRNA in JF1/Msf was increased compared with C3H/HeJ mice at postnatal (P) days P28 and P70. **b** Expression of lung *Fzd6* mRNA in JF1/Msf was decreased compared to C3H/HeJ mice at P28 and P70. Quantitative real-time polymerase chain reaction was performed, and the comparative cycle number threshold (C_T_) method (ΔΔC_T_) was used [ΔC_T_ = C_T_ (gene)-C_T_(*Actb*)]. Data are presented as expression relative to time-matched C3H/HeJ level (means ± SE; *n = 5* mice/strain/stage). *Significantly different from C3H/HeJ (ANOVA followed by all pairwise multiple-comparison procedures with Holm-Sidak method, *P <* 0.05). **c** Western blot analysis of CHIL3 from total lung homogenate of P28 C3H/HeJ and JF1/MsJ mice (*n* = 3 mice/strain; female). Lung transcript expression showed > 15 fold decreased *Chil3* expression in Jf1/MsJ compared to C3H/HeJ. β-actin (ACTB) was used as the control. Photograph is representative of at least three observations

**Fig. 2:**
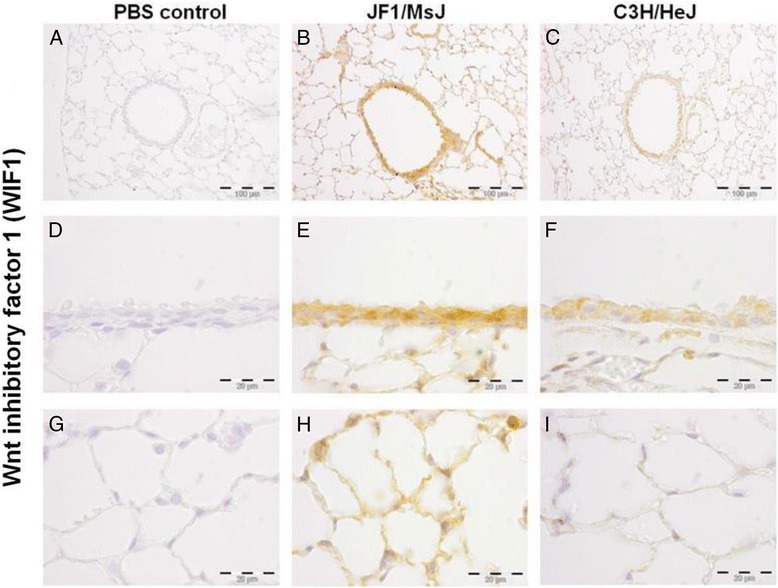
Localization of Wnt inhibitor factor 1 (WIF1) protein in the lungs of four weeks old female C3H/HeJ and JF1/MsJ mice (*n* = 5). Increased WIF1 immunostaining was detected in the **b, e** airway epithelium and **h** alveolar type I and type II cells of JF1/MsJ lungs compared with those of **c**, **f**, **i** C3H/HeJ mice. **a**, **d**, **g** Sections incubated phosphate-buffered saline (PBS control) without primary antisera

**Fig. 3:**
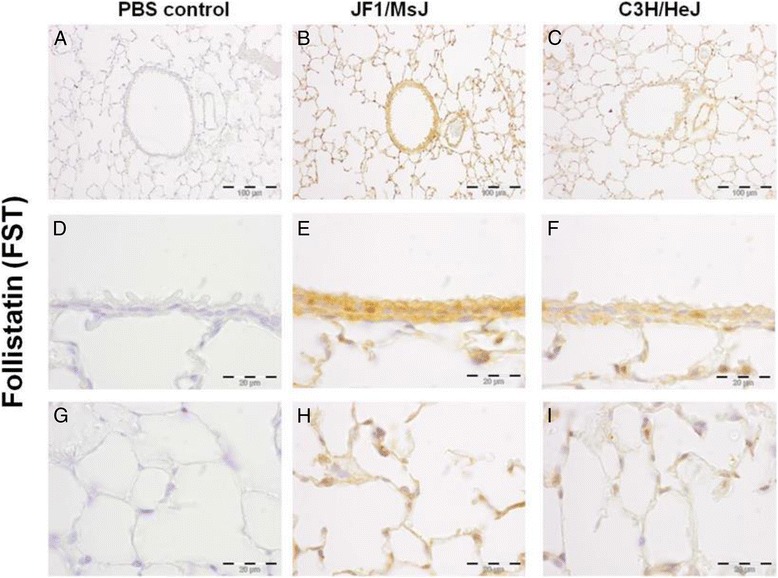
Localization of follistatin (FST) protein in the lungs of four weeks old female C3H/HeJ and JF1/MsJ mice (*n* = 5). Increased of FST immunostaining was detected in the **b**, **e** airways and **h** alveolar type I and type II cells of JF1/MsJ lungs compared with those of **c**, **f**, **i** C3H/HeJ mice. **a**, **d**, **g** Sections incubated phosphate-buffered saline (PBS control) without primary antisera

**Fig. 4:**
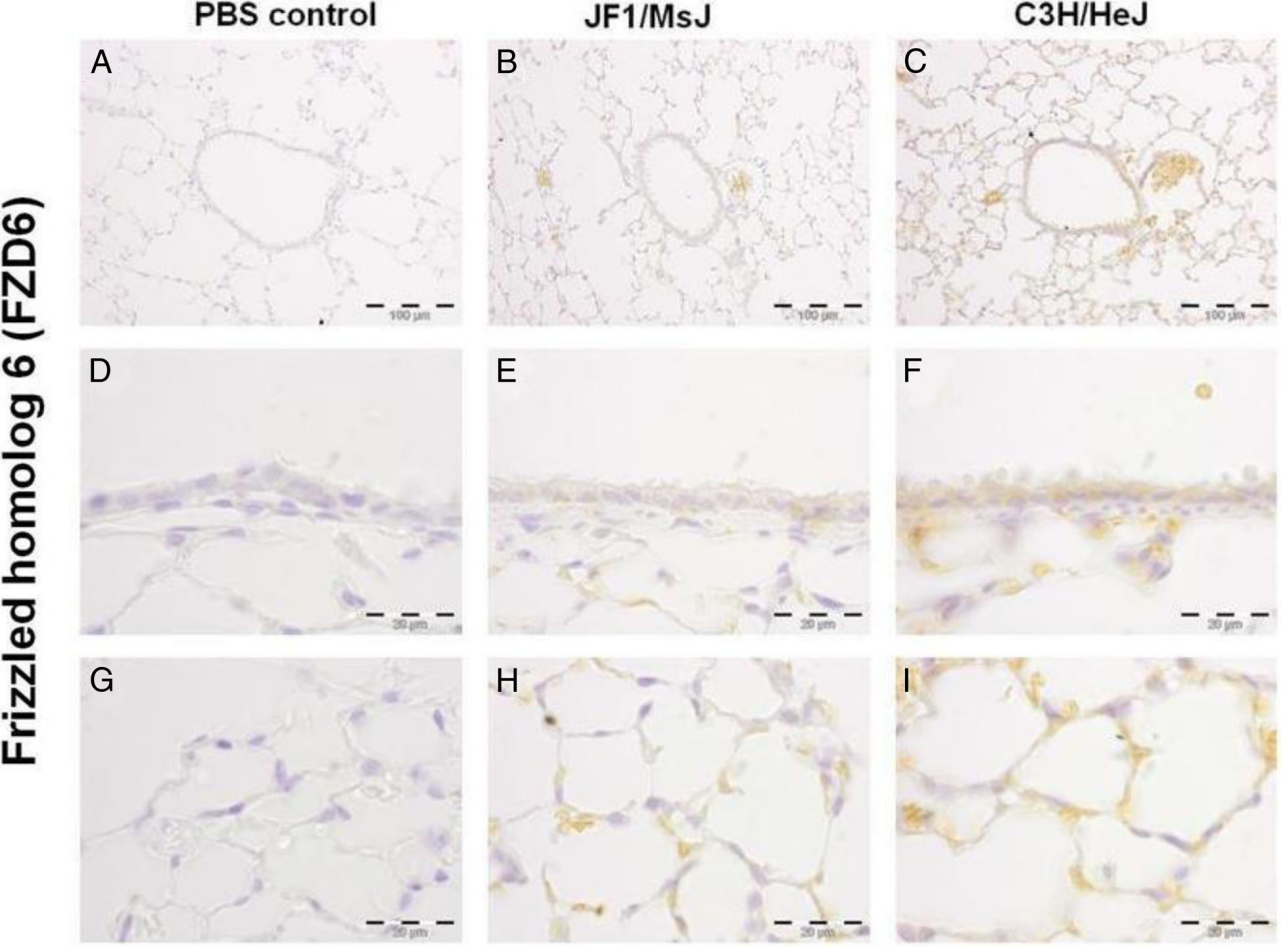
Localization of frizzled homolog 6 (FZD6) protein in the lungs of four weeks old female C3H/HeJ and JF1/MsJ mice (*n* = 5). Decreased FZD6 immunostaining was detected in the **b, e** airways and **h** alveolar type I and type II cells of JF1/MsJ lungs compared with those of **c**, **f**, **i** C3H/HeJ mice. **a**, **d**, **g** Sections incubated phosphate-buffered saline (PBS control) without primary antisera

Noteworthy transcripts altered in the later stages include several members of the wingless-type MMTV integration site family, member 1 (WNT1) protein family including increased Wnt inhibitor factor 1 (WIF1) and follistatin (FST) and decreased frizzled homolog 6 (FZD6) in JF1/MsJ compared to C3H/HeJ lungs. Decreased transcripts also included chitinase-like 1 (CHIL1), CHIL3, and hemolytic component (HC). qRT-PCR analysis of lung WIF1 was consistent with the microarray results (Fig. 1a, b). Western blot analysis for CHIL3 in the total lung protein homogenate was also consistent with the microarray results (Fig. 1c). Expression of the WNT pathway proteins (WIF1, FST and FZD6) were assessed by immunohistochemistry (Figs. 2, 3, 4). Increased immunoreactive WIF1 (Fig. 2) and FST (Fig. 3) were detected in the alveolar epithelial type I and II cells, bronchial epithelial cells, and macrophages of JF1/MsJ compared to C3H/HeJ mice. Decreased immunoreactive FZD6 was detected in the bronchial epithelial cells and endothelial cells of JF1/MsJ compared to C3H/HeJ mice (Fig. 4).

### Expression quantitative trait loci (eQTL) analysis

To further assess candidate genes for Lfnq3 and Lfnq4 associated with TLC, we compared JF1/MsJ to C3H/HeJ lung transcript levels at any of the three investigated stages. Employing this strategy, we identified 9 eQTLs located on mCh15 (Lfnq3) and mCh17 (Lfnq4) (Table 4) not previously reported for lung function development in mice. The candidate for Lfnq3 was leucine rich repeat containing 6 (testis) (*Lrrc6*). Several candidates for Lfnq4 were identified that include radial spoke head 1 homolog (Chlamydomonas) (*Rsph1*), formyl peptide receptor 1 (*Fpr1*), ATP-binding cassette, sub-family G (WHITE), member 1 (*Abcg1*), 3 histocompatibility 2 genes including class II antigen E beta (*H2-Eb1*), D region locus 1 (*H2-D1*), Q region locus 4 (*H2-Q4*), surfactant associated 2 (*Sfta2*), and gamma-aminobutyric acid (GABA) B receptor, 1 (*Gabbr1*).

**Table 4:** Candidate genes for total lung capacity (TLC) in mice identified through the expression quantitative trait loci (e-QTL) strategy comparing C3H/HeJ (C3H) and JF1/MsJ (JF1). [cut off for fold change ≥2.0 fold at least in one stage among embryonic stage 18, postnatal stage (P) 28 and P70; false discovery rate = 0.10]

Trait	QTL Region	QTL Location (Basepair)	Gene Description	Gene symbol/location (basepair)	EntrezID	Fold Difference		
						E18	P28	P70
Lfnq3 Chromosome: 15								
TLC, TLC/Bw	*D15Mit85 to D15Mit105*	40,313,626–72,500,731	Leucine rich repeat containing 6 (testis)	*Lrrc6* (66379858–66,500,910)	54,562	−2.03	ns	ns
Lfnq4 Chromosome: 17								
TLC	*D17Mit156 to D17Mit234*	10,987,597–39,053,168	Radial spoke head 1 homolog (Chlamydomonas)	*Rsph1* (31255019–31,277,356)	22,092	ns	2.81	2.63
			Formyl peptide receptor 1	*Fpr1* (17876471–17,883,939)	14,293	−2.05	−1.75	−2.26
			ATP-binding cassette, sub-family G (WHITE), member 1	*Abcg1* (31057694–31,117,984)	11,307	ns	−1.76	−2.44
			Surfactant associated 2	*Sfta2* (35649708–35,650,569)	433,102	−4.02	−2.19	−2.23
			Histocompatibility 2, class II antigen E beta	*H2-Eb1* (34305867–34,316,674)	14,969	ns	−2.23	−2.21
			Histocompatibility 2, D region locus 1	*H2-D1* (35263094–35,267,497)	14,964	ns	2.21	2.11
			Histocompatibility 2, Q region locus 4	*H2-Q4* (35379555–35,384,674)	15,015	−1.52	−3.19	−2.86
			Gamma-aminobutyric acid (GABA) B receptor, 1	*Gabbr1* (37045966–37,074,305)	54,393	−2.20	−1.68	−1.62

*ns* not significant

## Discussion

Hindered lung development results in failure to attain peak lung function by early adulthood that, in turn, may increase susceptibility to lung disease. In humans, lung development begins at gestational week 3 and alveolarization continues until at least age 5 years [37]. In mice, lung development begins E9.5 and continues up to P28 and alveolar septal formation continues up to P36 [38]. Recently, Beauchemin et al. [39] described 4-stage postnatal alveolarization with episodic transcriptional activity of pulmonary vascular genes in mice. The processes controlling lung development and growth are believed to be recapitulated following lung injury as genetic subroutines for repair and remodeling processes [40, 41]. Therefore, it is plausible that individuals having impaired lung development and growth that do not result in clinically significant symptom may have inefficient repair processes thereby predisposing them to subsequent chronic lung diseases. In this context, elucidating the genomics of lung function development through dissecting the genetics of lung development and growth is a promising approach to identify the predisposing factors. Thus, in this study, we sought to identify candidate genes for TLC by comparing the lung transcript profile of JF1/MsJ and C3H/HeJ strains at 3 critical times involving late embryonic, adolescent, and adult lung development.

The lung architectural differences detected in JF1/MsJ and C3H/HeJ mice are suggestive of their difference in development, growth and maturation events. JF1/MsJ mice have decreased mean chord length and mean alveolar volume but increased alveolar number density compared to C3H/HeJ. These findings suggest that JF1/MsJ mice with smaller alveoli (decreased mean chord length and alveolar volume) compensated by increased alveolar number density. Thus, the lungs of both strains exhibit near equal total alveolar surface area for gas exchange. Lung architectural differences detected between these mouse strains are consistent with their functional characteristics. Increased specific C_L-saline/air_ ratio in JF1/MsJ mice compared to C3H/HeJ suggests that surface tension contributes more than the elastic recoiling properties of JF1/MsJ lung in determining the lung compliance. This is in agreement with the smaller alveoli in JF1/MsJ mice. Increased elastin to collagen ratio and the decreased C_L-Air_/TLC and C_L-Saline_/TLC in JF1/MsJ mice indicates that elastic recoil due to tissue properties is higher in JF1/MsJ mice compared to C3H/HeJ.

In this study, we compared the lung transcript expression contrasting JF1/MsJ and C3H/HeJ mice at 3 stages, (I) embryonic day 18 (completion of embryonic lung development), (II) P28 (adolescent lung, bulk alveolar formation completed); and (III) P70 (mature lung). Noteworthy transcripts with consistent expression across the stages examined included those encoded by Wnt pathway and chitinase-like genes. Gene ontology cellular component “extracellular region” was enriched among decreased JF1/MsJ transcripts. We employed an e-QTL strategy and consistent expression to identify novel candidate genes for lung function development. e-QTL genes previously not reported with TLC development include *Lrrc6, Fpr1*, *Abcg1*, *Rsph1*, *H2-Eb1*, *H2-*D1, *H2-Q4*, *Sfta2*, and *Gabbr1*.

WIF1 inhibits extracellular WNT signaling that plays a crucial role during the lung development. Loss of SMAD1 transcriptional activation of *Wif1* has been associated with its decreased expression and increased Wnt/β-catenin signalling, resulting in abnormal distal lung epithelial cell differentiation and branching morphogenesis [42]. Reduced SMAD1 and WIF1 expression in nitrofen-induced hypoplastic lung resulted in developmental retardation during the saccular stage [43]. Our findings of increased lung WIF1 (>5-fold) transcript and protein levels in alveolar epithelial cells type I and II, as well as in bronchial epithelial cells during alveolar development in JF1/MsJ mice compared to C3H/HeJ strongly supports it to be a candidate for pulmonary function development.

Consistent with WIF1 expression, FST is also increased (>4-fold) in JF1/MsJ lungs compared to C3H/HeJ. Canonical Wnt/β-catenin signalling controls FST signalling in satellite cell derived myoblasts [44]. FST-deficient mice exhibit severely retarded overall growth, breathing failure, and perinatal death with fluid filled lungs and poorly expanded alveolar spaces [45]. FST binds and inhibits activins, which are members of the transforming growth factor beta superfamily that plays a significant role in the lung developmental processes [46]. Therapeutic intervention with FST markedly reduced the number of infiltrating cells and ameliorated the destruction of lung architecture in bleomycin-treated rats [47].

FZD6 also functions as a negative regulator of the canonical Wnt/β-catenin signaling cascade. FZD6 controls macroscopic hair patterning in mice and studies show epithelial cells as the source of FZD6-dependent signaling [48]. Wnt signaling pathway was enriched for annotation in the murine Developing Lung Characteristic Subtranscriptome (mDLCS) involving C57BL/6 J, A/J and C3H/HeJ inbred strains. WIF1 and FZD6 transcripts also expressed significant strain dependent modulation during alveolar development [39].

The proteins encoded by *Chil1* (aka *Chi3i1* or *YKL40*) and *Chil3* (aka *Chi3l3* or *Ym1*) are similar to bacterial chitinases but lacks chitinase activity. JF1/MsJ mice with lower basal lung function have decreased lung CHIL1 (>2 fold) and CHIL3 (>15 fold) transcripts. In humans, the gene homolog for mouse *Chil1* is *CHIL1*. Variations in *CHIL1* have been associated with circulating and airway CHIL1 protein levels along with asthma prevalence, severity, hospital admissions, and lung function in children and young adults [49–53]. Variations in *CHIL1* are also considered to modulate age-adjusted lung function in cystic fibrosis patients [54]. Elevated serum CHIL1 protein is associated with severe persistent asthma among adults but not in children [55]. However, increased CHIL1 levels are detected among children with severe, steroid resistant asthma [56]. Serum CHIL1 levels are negatively correlated with percent FEV_1_ while positively correlated with low attenuation area/total lung area percent in COPD patients [57]. Elevated CHIL1 protein has been detected in bronchoalveolar lavage fluid and sputum, with higher numbers of CHIL1-expressing cells in bronchial biopsies of smokers with COPD [58, 59]. CHIL1 can activate Wnt/β-catenin signaling in alveolar macrophage [60].

Amino acid sequence homology of CHIL3 to proteins associated with tissue remodeling together with their binding capacity with heparin sulfate and GlcN oligomers suggests their role in airway wall remodeling in the allergic lung [61]. CHIL3 protein is abundantly expressed in the allergic mouse lung and enhances Th2 cytokine production by inhibiting 12/15(S)-lipoxygenase which generates lipid metabolites that interferes with T cell proliferation [62]. However, a homologous sequence to *Chil3* has not been found in the human genome.

The evaluation of eQTL located in Lfnq3 and Lfnq4 provided 9 candidate genes worthy of further analysis for their possible role in lung development and growth. Located in Lfnq3 region linked to TLC on mCh15, *Lrrc6 is* a gene expressed in the respiratory epithelium that is essential for proper cilia axonemal assembly of inner and outer dynein arms. Kott and colleagues identified an early frameshift in *LRRC6* that is associated with primary ciliary dyskinesia (PCD) [63]. PCD is a group of autosomal-recessive developmental disorders resulting from cilia and sperm-flagella defects, which can lead to respiratory infections, situs inversus, and male infertility [64]. In zebrafish, *lrrc6 (aka seahorse)* mutants produce ventral body curvature and kidney cysts [65, 66] and LRRC6 protein associates with disheveled, which constrains the canonical Wnt/β-catenin pathway and promotes the non-canonical Wnt pathway during gastrulation [67]. In gene-targeted mice lacking functional LRRC6, cilia microtubules remained normal but the outer dynein arms (ODAs), the structures essential for the ciliary beating, are absent. In the absence of LRRC6, ODA proteins that normally are assembled in the cytoplasm and transported to the ciliary axoneme, remain in the cytoplasm and are not transported to the ciliary axoneme [68, 69].

Of the eight differentially expressed transcripts located within the Lfnq4 region in mCh17 that has been linked to TLC, *Rsph1*, *Fpr1*, *Abcg1*, and *Sfta2* are most noteworthy. Like *LRRC6* variants, splice site (275-2A > C) and non-sense (433 C > T) polymorphisms in *RSPH1* are associated with PCD [64]. Although persons with these variants can have unexplained neonatal respiratory distress [70], the splice site variation in RSPH1 is associated with a lower prevalence of neonatal respiratory distress, later onset of daily wet cough, and better adult lung function than other forms of PCD [71].

Mitochondrial damage-associated molecular patterns (DAMPs) include formyl peptides that activate human neutrophils through FPR1. On human neutrophils, FPR1 interacts with annexin A1 (aka lipocortin I), which mediated anti-inflammatory activities of glucocorticoids and can regulate epidermal growth factor receptor (EGFR) localization and activity [72, 73]. Gene-targeted *Fpr1* ^*−/−*^ mice have a slight, but significant decrease in mean chord length compared to strain-matched (C57BL/6 J) control mice [74]. In addition, *Fpr1* ^*−/−*^ mice have decreased inflammation and emphysema compared to control mice after cigarette smoke exposure. In humans, cigarette smoking increases the number of FPRs on the surface of neutrophils, which is greater in smokers with COPD than smokers without COPD [75, 76]. In addition, a variant rs867228 (minor allele frequency = 0.21) producing a loss-of-function allele in *FPR1* is associated with poor metastasis-free and overall survival in breast and colorectal cancer patients receiving adjuvant chemotherapy [77].

Patients with pulmonary alveolar proteinosis (PAP) display impaired surfactant clearance, foamy, lipid-filled alveolar macrophages, and increased cholesterol metabolites within the lung. Also regulated by Wnt/β-catenin signalling [78], ABCG1 lipid transporter is considered to be a key downstream target of colony stimulating factor 2 (aka GM-CSF) and is necessary for proper surfactant catabolism [79, 80]. Mice lacking ABCG1 develop alveolar type II cell hypertrophy with lipid deposition, increased levels of surfactant [81], and develop more severe lung fibrosis after bleomycin [82]. Compared with strain-matched control mice, the lungs of ABCG1 null mice are inflamed with macrophage accumulation, lymphocytic infiltration, hemorrhage, eosinophilic crystals, and elevated levels of cytokines and cytokine receptors. BALF samples obtained from *Abcg1* ^*−/−*^ mice are marked by increased foamy macrophages and leukocytes and the presence of multiple markers of inflammation including crystals of CHIL3 protein [83]. In mice, ABCG1 is also required for pulmonary B-1 B cell and natural antibody homeostasis [84]. Variations in *ABCG1* (rs13050646) and *HLA-A* (rs3823343, rs2517725) have been associated to IgE dysregulation and atopy [85].

SFTA2 is a secretory protein predominantly expressed by alveolar type II cells and non-ciliated bronchiolar cells [86]. Similar to hydrophobic surfactant proteins B (SFTB) and SFTPC, SFTA2 protein may share particular physicochemical properties [87] and lung SFTA2 mRNA deceases in mice treated with lipopolysaccharide [86]. However, unlike SFTFB and SFTPC, SFTA2 does not co-localize to lamellar bodies, but is found to co-localize to the Golgi and clathrin vesicles like hydrophilic SFTPA1 and SFTPD [86]. Little is known about the disease phenotypes associated with SFTA2 variants, but human *SFTA2* is located in a chromosomal locus associated with diffused panbronchiolitis.

Recent studies have implicated Wnt/β-catenin pathway to play an important role in alveolar development [39]. In an attempt to summarize the plausible effect of altered expression of the identified candidate genes based on the above reviewed information, our data suggest that Wnt/β-catenin signaling may be altered during postnatal lung development and growth of JF1/MsJ as compared to C3H/HeJ mice. This is supported by the increased WIF1 transcript and protein levels in JF1/MsJ lungs. Primary ciliary proteins, like LRRC6 and RSPH, are required not only for regulation of Wnt/β-catenin signaling, but also act as downstream effectors of the Wnt/β-catenin pathway. [88] Alterations in a chitinase-like protein, CHIL1, an activin-binding protein, FST, and a lipid transporter, ABCG1, also implicates this pathway. However, elucidation of the precise mode of action of genetic variants in these genes in determining lung function development warrants functional and mechanistic investigations.

## Conclusions

This study demonstrates that the divergent pulmonary function between C3H/HeJ and JF1/MsJ mice is associated with differences in transcription expression profiles in lung. We further dissected the altered genetic signature of divergent lung function development among C3H/HeJ and JF1/MsJ mice and identified several novel genes, especially those with roles in Wnt/β-catenin signalling, not previously associated to lung function. The study also provides a reference of transcripts altered between mouse strains with divergent TLC at the completion of embryonic lung development, bulk alveolar formation and lung growth. The generated data will provide a point base for future association, functional and mechanistic studies for pulmonary function.

## Additional file

Additional file 1: Table S1. Increased lung transcripts in JF1/MsJ (JF1) compared to C3H/HeJ (C3H) mice at embryonic day 18. **Table S2.** Decreased lung transcripts in JF1/MsJ (JF1) compared to C3H/HeJ (C3H) mice at embryonic stage 18. **Table S3.** Increased lung transcripts in JF1/MsJ (JF1) compared to C3H/HeJ (C3H) mice at postnatal day 28. **Table S4.** Decreased lung transcripts in JF1/MsJ (JF1) compared to C3H/HeJ (C3H) mice at postnatal day 28. **Table S5.** Increased lung transcripts in JF1/MsJ (JF1) compared to C3H/HeJ (C3H) mice at postnatal day 70. **Table S6.** Decreased lung transcripts in JF1/MsJ (JF1) compared to C3H/HeJ (C3H) mice at postnatal day 70. **Table S7.** Transcripts showing consistent pattern of expression lung transcripts in JF1/MsJ (JF1) compared to C3H/HeJ (C3H) mice across E18/ P28 stages. **Table S8.** Transcripts showing consistent pattern of expression lung transcripts in JF1/MsJ (JF1) compared to C3H/HeJ (C3H) mice across P28/ P70 stages. **Figure S1.** Representative lung sections showing smaller alveoli in JF1/Msf (JF1) mice compared to C3H/HeJ (C3H) in both males and females. (DOCX 216 kb)

## Abbreviations

*Abcg1*: ATP-binding cassette, sub-family G (WHITE), member 1
ACTB: Beta actin
BALF: Bronchoalveolar lavage fluid
*Chil*: Chitinase-like 1
*Chil3*: Chitinase-like 3
C_L_: Compliance
COPD: Chronic obstructive pulmonary disease
E: Embryonic day
e-QTL: Expression-quantitative trait loci
FEV_1_: Forced expiratory volume in 1 s
*Fpr1*: Formyl peptide receptor 1
*Fst*: Follistatin
*Fzd6*: Frizzled homolog 6
*Gabbr1*: Gamma-aminobutyric acid (GABA) B receptor, 1
GO: Gene ontology
*H2-D1*: Histocompatibility 2, D region locus 1
*H2-Eb1*: Histocompatibility 2, class II antigen E beta
*H2-Q4*: Histocompatibility 2, Q region locus 4
IAEC: Institutional Animal Ethics Committee
ip: Intra-peritoneal
*Kit*: *Kit* oncogene
Lfnq: Lung function quantitative trait loci
*Lrrc6*: Leucine rich repeat containing 6 (testis)
mCh: Mouse chromosome
mDLCS: Developing lung characteristic subtranscriptome
P: Postnatal day
qRT-PCR: Quantitative real time polymerase chain reaction
QTL: Quantitative trait loci
*Rsph1*: Radial spoke head 1 homolog (Chlamydomonas)
*Sfta2*: Surfactant associated 2
*Sod3*: Superoxide dismutase 3, extracellular
*Spp1*: Secreted phosphoprotein 1
SUR: Systematic uniform random
TEM: Transmission electron microscope
TLC: Total lung capacity
*Wif1*: Wnt inhibitor factor 1

## References

[1] Krauss-Etschmann S, Bush A, Bellusci S, Brusselle GG, Dahlen SE, Dehmel S, Eickelberg O, Gibson G, Hylkema MN, Knaus P, Konigshoff M, Lloyd CM, Macciarini P, Mailleux A, Marsland BJ, Postma DS, Roberts G, Samakovlis C, Stocks J, Vandesompele J, Wjst M, Holloway J (2013). Of flies, mice and men: a systematic approach to understanding the early life origins of chronic lung disease. Thorax.

[2] Stocks J, Hislop A, Sonnappa S (2013). Early lung development: lifelong effect on respiratory health and disease. Lancet Respir Med.

[3] Stocks J, Sonnappa S (2013). Early life influences on the development of chronic obstructive pulmonary disease. Ther Adv Respir Dis.

[4] Martinez FD (2009). The origins of asthma and chronic obstructive pulmonary disease in early life. Proc Am Thorac Soc.

[5] Lange P, Celli B, Agusti A, Boje Jensen G, Divo M, Faner R, Guerra S, Marott JL, Martinez FD, Martinez-Camblor P, Meek P, Owen CA, Petersen H, Pinto-Plata V, Schnohr P, Sood A, Soriano JB, Tesfaigzi Y, Vestbo J (2015). Lung-Function Trajectories Leading to Chronic Obstructive Pulmonary Disease. N Engl J Med.

[6] Repapi E, Sayers I, Wain LV, Burton PR, Johnson T, Obeidat M, Zhao JH, Ramasamy A, Zhai G, Vitart V, Huffman JE, Igl W, Albrecht E, Deloukas P, Henderson J, Granell R, WL MA, Rudnicka AR, Barroso I, Loos RJ, Wareham NJ, Mustelin L, Rantanen T, Surakka I, Imboden M, Wichmann HE, Grkovic I, Jankovic S, Zgaga L, Hartikainen AL, Peltonen L, Gyllensten U, Johansson A, Zaboli G, Campbell H, Wild SH, Wilson JF, Glaser S, Homuth G, Volzke H, Mangino M, Soranzo N, Spector TD, Polasek O, Rudan I, Wright AF, Heliovaara M, Ripatti S, Pouta A, Naluai AT, Olin AC, Toren K, Cooper MN, James AL, Palmer LJ, Hingorani AD, Wannamethee SG, Whincup PH, Smith GD, Ebrahim S, TM MK, Pavord ID, AK ML, Morris AD, Porteous DJ, Cooper C, Dennison E, Shaheen S, Karrasch S, Schnabel E, Schulz H, Grallert H, Bouatia-Naji N, Delplanque J, Froguel P, Blakey JD, NRS T, Britton JR, Morris RW, Holloway JW, Lawlor DA, Hui J, Nyberg F, Jarvelin MR, Jackson C, Kahonen M, Kaprio J, Probst-Hensch NM, Koch B, Hayward C, Evans DM, Elliott P, Strachan DP, Hall IP, Tobin MD, Wellcome Trust Case Control C (2010). Genome-wide association study identifies five loci associated with lung function. Nat Genet.

[7] Yao TC, Du G, Han L, Sun Y, Hu D, Yang JJ, Mathias R, Roth LA, Rafaels N, Thompson EE, Loisel DA, Anderson R, Eng C, Arruabarrena Orbegozo M, Young M, Klocksieben JM, Anderson E, Shanovich K, Lester LA, Williams LK, Barnes KC, Burchard EG, Nicolae DL, Abney M, Ober C (2014). Genome-wide association study of lung function phenotypes in a founder population. J Allergy Clin Immunol.

[8] Hancock DB, Eijgelsheim M, Wilk JB, Gharib SA, Loehr LR, Marciante KD, Franceschini N, van Durme YM, Chen TH, Barr RG, Schabath MB, Couper DJ, Brusselle GG, Psaty BM, van Duijn CM, Rotter JI, Uitterlinden AG, Hofman A, Punjabi NM, Rivadeneira F, Morrison AC, Enright PL, North KE, Heckbert SR, Lumley T, Stricker BH, O'Connor GT, London SJ (2010). Meta-analyses of genome-wide association studies identify multiple loci associated with pulmonary function. Nat Genet.

[9] Soler Artigas M, Loth DW, Wain LV, Gharib SA, Obeidat M, Tang W, Zhai G, Zhao JH, Smith AV, Huffman JE, Albrecht E, Jackson CM, Evans DM, Cadby G, Fornage M, Manichaikul A, Lopez LM, Johnson T, Aldrich MC, Aspelund T, Barroso I, Campbell H, Cassano PA, Couper DJ, Eiriksdottir G, Franceschini N, Garcia M, Gieger C, Gislason GK, Grkovic I, Hammond CJ, Hancock DB, Harris TB, Ramasamy A, Heckbert SR, Heliovaara M, Homuth G, Hysi PG, James AL, Jankovic S, Joubert BR, Karrasch S, Klopp N, Koch B, Kritchevsky SB, Launer LJ, Liu Y, Loehr LR, Lohman K, Loos RJ, Lumley T, Al Balushi KA, Ang WQ, Barr RG, Beilby J, Blakey JD, Boban M, Boraska V, Brisman J, Britton JR, Brusselle GG, Cooper C, Curjuric I, Dahgam S, Deary IJ, Ebrahim S, Eijgelsheim M, Francks C, Gaysina D, Granell R, Gu X, Hankinson JL, Hardy R, Harris SE, Henderson J, Henry A, Hingorani AD, Hofman A, Holt PG, Hui J, Hunter ML, Imboden M, Jameson KA, Kerr SM, Kolcic I, Kronenberg F, Liu JZ, Marchini J, McKeever T, Morris AD, Olin AC, Porteous DJ, Postma DS, Rich SS, Ring SM, Rivadeneira F, Rochat T, Sayer AA, Sayers I, Sly PD, Smith GD, Sood A, Starr JM, Uitterlinden AG, Vonk JM, Wannamethee SG, Whincup PH, Wijmenga C, Williams OD, Wong A, Mangino M, Marciante KD, WL MA, Meibohm B, Morrison AC, North KE, Omenaas E, Palmer LJ, Pietilainen KH, Pin I, Pola Sbreve Ek O, Pouta A, Psaty BM, Hartikainen AL, Rantanen T, Ripatti S, Rotter JI, Rudan I, Rudnicka AR, Schulz H, Shin SY, Spector TD, Surakka I, Vitart V, Volzke H, Wareham NJ, Warrington NM, Wichmann HE, Wild SH, Wilk JB, Wjst M, Wright AF, Zgaga L, Zemunik T, Pennell CE, Nyberg F, Kuh D, Holloway JW, Boezen HM, Lawlor DA, Morris RW, Probst-Hensch N, Kaprio J, Wilson JF, Hayward C, Kahonen M, Heinrich J, Musk AW, Jarvis DL, Glaser S, Jarvelin MR, Ch Stricker BH, Elliott P, O'Connor GT, Strachan DP, London SJ, Hall IP, Gudnason V, Tobin MD, International Lung Cancer C, Consortium G (2011). Genome-wide association and large-scale follow up identifies 16 new loci influencing lung function. Nat Genet.

[10] Soler Artigas M, Wain LV, Repapi E, Obeidat M, Sayers I, Burton PR, Johnson T, Zhao JH, Albrecht E, Dominiczak AF, Kerr SM, Smith BH, Cadby G, Hui J, Palmer LJ, Hingorani AD, Wannamethee SG, Whincup PH, Ebrahim S, Smith GD, Barroso I, Loos RJ, Wareham NJ, Cooper C, Dennison E, Shaheen SO, Liu JZ, Marchini J, Dahgam S, Naluai AT, Olin AC, Karrasch S, Heinrich J, Schulz H, TM MK, Pavord ID, Heliovaara M, Ripatti S, Surakka I, Blakey JD, Kahonen M, Britton JR, Nyberg F, Holloway JW, Lawlor DA, Morris RW, James AL, Jackson CM, Hall IP, Tobin MD, Medical Research Council National Survey of H, Development Respiratory Study T, SpiroMeta C (2011). Effect of five genetic variants associated with lung function on the risk of chronic obstructive lung disease, and their joint effects on lung function. Am J Respir Crit Care Med.

[11] Obeidat M, Hao K, Bosse Y, Nickle DC, Nie Y, Postma DS, Laviolette M, Sandford AJ, Daley DD, Hogg JC, Elliott WM, Fishbane N, Timens W, Hysi PG, Kaprio J, Wilson JF, Hui J, Rawal R, Schulz H, Stubbe B, Hayward C, Polasek O, Jarvelin MR, Zhao JH, Jarvis D, Kahonen M, Franceschini N, North KE, Loth DW, Brusselle GG, Smith AV, Gudnason V, Bartz TM, Wilk JB, O'Connor GT, Cassano PA, Tang W, Wain LV, Soler Artigas M, Gharib SA, Strachan DP, Sin DD, Tobin MD, London SJ, Hall IP, Pare PD (2015). Molecular mechanisms underlying variations in lung function: a systems genetics analysis. Lancet Respir Med.

[12] Tang W, Kowgier M, Loth DW, Soler Artigas M, Joubert BR, Hodge E, Gharib SA, Smith AV, Ruczinski I, Gudnason V, Mathias RA, Harris TB, Hansel NN, Launer LJ, Barnes KC, Hansen JG, Albrecht E, Aldrich MC, Allerhand M, Barr RG, Brusselle GG, Couper DJ, Curjuric I, Davies G, Deary IJ, Dupuis J, Fall T, Foy M, Franceschini N, Gao W, Glaser S, Gu X, Hancock DB, Heinrich J, Hofman A, Imboden M, Ingelsson E, James A, Karrasch S, Koch B, Kritchevsky SB, Kumar A, Lahousse L, Li G, Lind L, Lindgren C, Liu Y, Lohman K, Lumley T, WL MA, Meibohm B, Morris AP, Morrison AC, Musk B, North KE, Palmer LJ, Probst-Hensch NM, Psaty BM, Rivadeneira F, Rotter JI, Schulz H, Smith LJ, Sood A, Starr JM, Strachan DP, Teumer A, Uitterlinden AG, Volzke H, Voorman A, Wain LV, Wells MT, Wilk JB, Williams OD, Heckbert SR, Stricker BH, London SJ, Fornage M, Tobin MD, O'Connor GT, Hall IP, Cassano PA (2014). Large-scale genome-wide association studies and meta-analyses of longitudinal change in adult lung function. PLoS One.

[13] Loth DW, Soler Artigas M, Gharib SA, Wain LV, Franceschini N, Koch B, Pottinger TD, Smith AV, Duan Q, Oldmeadow C, Lee MK, Strachan DP, James AL, Huffman JE, Vitart V, Ramasamy A, Wareham NJ, Kaprio J, Wang XQ, Trochet H, Kahonen M, Flexeder C, Albrecht E, Lopez LM, de Jong K, Thyagarajan B, Alves AC, Enroth S, Omenaas E, Joshi PK, Fall T, Vinuela A, Launer LJ, Loehr LR, Fornage M, Li G, Wilk JB, Tang W, Manichaikul A, Lahousse L, Harris TB, North KE, Rudnicka AR, Hui J, Gu X, Lumley T, Wright AF, Hastie ND, Campbell S, Kumar R, Pin I, Scott RA, Pietilainen KH, Surakka I, Liu Y, Holliday EG, Schulz H, Heinrich J, Davies G, Vonk JM, Wojczynski M, Pouta A, Johansson A, Wild SH, Ingelsson E, Rivadeneira F, Volzke H, Hysi PG, Eiriksdottir G, Morrison AC, Rotter JI, Gao W, Postma DS, White WB, Rich SS, Hofman A, Aspelund T, Couper D, Smith LJ, Psaty BM, Lohman K, Burchard EG, Uitterlinden AG, Garcia M, Joubert BR, WL MA, Musk AB, Hansel N, Heckbert SR, Zgaga L, van Meurs JB, Navarro P, Rudan I, Oh YM, Redline S, Jarvis DL, Zhao JH, Rantanen T, O'Connor GT, Ripatti S, Scott RJ, Karrasch S, Grallert H, Gaddis NC, Starr JM, Wijmenga C, Minster RL, Lederer DJ, Pekkanen J, Gyllensten U, Campbell H, Morris AP, Glaser S, Hammond CJ, Burkart KM, Beilby J, Kritchevsky SB, Gudnason V, Hancock DB, Williams OD, Polasek O, Zemunik T, Kolcic I, Petrini MF, Wjst M, Kim WJ, Porteous DJ, Scotland G, Smith BH, Viljanen A, Heliovaara M, Attia JR, Sayers I, Hampel R, Gieger C, Deary IJ, Boezen HM, Newman A, Jarvelin MR, Wilson JF, Lind L, Stricker BH, Teumer A, Spector TD, Melen E, Peters MJ, Lange LA, Barr RG, Bracke KR, Verhamme FM, Sung J, Hiemstra PS, Cassano PA, Sood A, Hayward C, Dupuis J, Hall IP, Brusselle GG, Tobin MD, London SJ (2014). Genome-wide association analysis identifies six new loci associated with forced vital capacity. Nat Genet.

[14] Young S, Sherrill DL, Arnott J, Diepeveen D, LeSouef PN, Landau LI (2000). Parental factors affecting respiratory function during the first year of life. Pediatr Pulmonol.

[15] Murray CS, Pipis SD, McArdle EC, Lowe LA, Custovic A, Woodcock A, National Asthma Campaign-Manchester A, Allergy Study G. Lung function at one month of age as a risk factor for infant respiratory symptoms in a high risk population. Thorax 2002;57: 388–392. 10.1136/thorax.57.5.388 PMC1746314 11978912

[16] Mullane D, Turner SW, Cox DW, Goldblatt J, Landau LI, le Souef PN (2013). Reduced infant lung function, active smoking, and wheeze in 18-year-old individuals. JAMA Pediatr.

[17] Turner S, Fielding S, Mullane D, Cox DW, Goldblatt J, Landau L, le Souef P (2014). A longitudinal study of lung function from 1 month to 18 years of age. Thorax.

[18] Skripak JM (2014). Persistent effects of maternal smoking during pregnancy on lung function and asthma in adolescents. Pediatrics.

[19] Reinhard C, Eder G, Fuchs H, Ziesenis A, Heyder J, Schulz H (2002). Inbred strain variation in lung function. Mamm Genome.

[20] Reinhard C, Meyer B, Fuchs H, Stoeger T, Eder G, Ruschendorf F, Heyder J, Nurnberg P, de Angelis MH, Schulz H (2005). Genomewide linkage analysis identifies novel genetic Loci for lung function in mice. Am J Respir Crit Care Med.

[21] Ganguly K, Stoeger T, Wesselkamper SC, Reinhard C, Sartor MA, Medvedovic M, Tomlinson CR, Bolle I, Mason JM, Leikauf GD, Schulz H (2007). Candidate genes controlling pulmonary function in mice: transcript profiling and predicted protein structure. Physiol Genomics.

[22] Ganguly K, Upadhyay S, Irmler M, Takenaka S, Pukelsheim K, Beckers J, De Angelis MH, Hamelmann E, Stoeger T, Schulz H (2011). Impaired resolution of inflammatory response in the lungs of JF1/Msf mice following carbon nanoparticle instillation. Respir Res.

[23] Leikauf GD, Concel VJ, Liu P, Bein K, Berndt A, Ganguly K, Jang AS, Brant KA, Dietsch M, Pope-Varsalona H, Dopico RA, Di YP, Li Q, Vuga LJ, Medvedovic M, Kaminski N, You M, Prows DR (2011). Haplotype association mapping of acute lung injury in mice implicates activin a receptor, type 1. Am J Respir Crit Care Med.

[24] Ganguly K, Upadhyay S, Irmler M, Takenaka S, Pukelsheim K, Beckers J, Hamelmann E, Schulz H, Stoeger T (2009). Pathway focused protein profiling indicates differential function for IL-1B, −18 and VEGF during initiation and resolution of lung inflammation evoked by carbon nanoparticle exposure in mice. Part Fibre Toxicol.

[25] Ganguly K, Depner M, Fattman C, Bein K, Oury TD, Wesselkamper SC, Borchers MT, Schreiber M, Gao F, von Mutius E, Kabesch M, Leikauf GD, Schulz H (2009). Superoxide dismutase 3, extracellular (SOD3) variants and lung function. Physiol Genomics.

[26] Ganguly K, Martin TM, Concel VJ, Upadhyay S, Bein K, Brant KA, George L, Mitra A, Thimraj TA, Fabisiak JP, Vuga LJ, Fattman C, Kaminski N, Schulz H, Leikauf GD (2014). Secreted phosphoprotein 1 is a determinant of lung function development in mice. Am J Respir Cell Mol Biol.

[27] Lindsey JY, Ganguly K, Brass DM, Li Z, Potts EN, Degan S, Chen H, Brockway B, Abraham SN, Berndt A, Stripp BR, Foster WM, Leikauf GD, Schulz H, Hollingsworth JW (2011). c-Kit is essential for alveolar maintenance and protection from emphysema-like disease in mice. Am J Respir Crit Care Med.

[28] Hsia CC, Hyde DM, Ochs M, Weibel ER, Structure AEJTFoQAoL (2010). An official research policy statement of the American Thoracic Society/European Respiratory Society: standards for quantitative assessment of lung structure. Am J Respir Crit Care Med.

[29] Fehrenbach H, Zimmermann G, Starke E, Bratu VA, Conrad D, Yildirim AO, Fehrenbach A (2007). Nitrogen dioxide induces apoptosis and proliferation but not emphysema in rat lungs. Thorax.

[30] Madurga A, Mizikova I, Ruiz-Camp J, Vadasz I, Herold S, Mayer K, Fehrenbach H, Seeger W, Morty RE (2014). Systemic hydrogen sulfide administration partially restores normal alveolarization in an experimental animal model of bronchopulmonary dysplasia. Am J Physiol Lung Cell Mol Physiol.

[31] Fehrenbach H, Ochs M, Uhlig S, Taylor A (1998). Studying lung ultrastructure. Methods in pulmonary research.

[32] Haidar A, Ryder TA, Mobberley MA, Wigglesworth JS (1992). Two techniques for electron opaque staining of elastic fibres using tannic acid in fresh and formalin fixed tissue. J Clin Pathol.

[33] Schulz H, Johner C, Eder G, Ziesenis A, Reitmeier P, Heyder J, Balling R (2002). Respiratory mechanics in mice: strain and sex specific differences. Acta Physiol Scand.

[34] R Development Core team. R: A language and environment for statistical computing. Vienna: the R Foundation for Statistical Computing; 2011.

[35] Rainer J, Sanchez-Cabo F, Stocker G, Sturn A, Trajanoski Z (2006). CARMA web: comprehensive R- and Bioconductor-based web service for microarray data analysis. Nucleic Acids Res.

[36] Huang DW, Sherman BT, Lempicki RA (2009). Systematic and integrative analysis of large gene lists using DAVID Bioinformatics Resources. Nature Protoc.

[37] Warburton D, El-Hashash A, Carraro G, Tiozzo C, Sala F, Rogers O, De Langhe S, Kemp PJ, Riccardi D, Torday J, Bellusci S, Shi W, Lubkin SR, Jesudason E, Koopman P (2010). Lung organogenesis. Organogenesis in development.

[38] Schittny JC, Mund SI, Stampanoni M (2008). Evidence and structural mechanism for late lung alveolarization. Am J Physiol Lung Cell Mol Physiol.

[39] Beauchemin KJ, Wells JM, Kho AT, Philip VM, Kamir D, Kohane IS, Graber JH, Bult CJ (2016). Temporal dynamics of the developing lung transcriptome in three common inbred strains of laboratory mice reveals multiple stages of postnatal alveolar development. PeerJ.

[40] Hagood JS, Ambalavanan N (2013). Systems biology of lung development and regeneration: current knowledge and recommendations for future research. Wiley Interdiscip Rev Syst Biol Med.

[41] Stabler CT, Morrisey EE (2017). Developmental pathways in lung regeneration. Cell Tissue Res.

[42] Xu B, Chen C, Chen H, Zheng SG, Bringas P, Xu M, Zhou X, Chen D, Umans L, Zwijsen A, Shi W (2011). Smad1 and its target gene Wif1 coordinate BMP and Wnt signaling activities to regulate fetal lung development. Development.

[43] Fujiwara N, Doi T, Gosemann JH, Kutasy B, Friedmacher F, Puri P (2012). Smad1 and WIF1 genes are downregulated during saccular stage of lung development in the nitrofen rat model. Pediatr Surg Int.

[44] Jones AE, Price FD, Le Grand F, Soleimani VD, Dick SA, Megeney LA, Rudnicki MA (2015). Wnt/beta-catenin controls follistatin signalling to regulate satellite cell myogenic potential. Skelet Muscle.

[45] Matzuk MM, Lu N, Vogel H, Sellheyer K, Roop DR, Bradley A (1995). Multiple defects and perinatal death in mice deficient in follistatin. Nature.

[46] Zhao Y, Silbajoris R, Young SL (1996). Identification and developmental expression of two activin receptors in baboon lung. Biochem Biophys Res Commun.

[47] Aoki F, Kurabayashi M, Hasegawa Y, Kojima I (2005). Attenuation of bleomycin-induced pulmonary fibrosis by follistatin. Am J Respir Crit Care Med.

[48] Guo N, Hawkins C, Nathans J (2004). Frizzled6 controls hair patterning in mice. Proc Natl Acad Sci U S A.

[49] Tang H, Fang Z, Sun Y, Li B, Shi Z, Chen J, Zhang T, Xiu Q (2010). YKL-40 in asthmatic patients, and its correlations with exacerbation, eosinophils and immunoglobulin E. Eur Respir J.

[50] Cunningham J, Basu K, Tavendale R, Palmer CN, Smith H, Mukhopadhyay S (2011). The CHI3L1 rs4950928 polymorphism is associated with asthma-related hospital admissions in children and young adults. Ann Allergy Asthma Immunol.

[51] Verlaan DJ, Ouimet M, Adoue V, Sirois-Gagnon D, Lariviere M, Ge B, Beaulieu P, Dias J, Lam KC, Koka V, Laprise C, Pastinen T, Sinnett D (2012). Promoter polymorphisms in CHI3L1 are associated with asthma. J Allergy Clin Immunol.

[52] Gomez JL, Crisafi GM, Holm CT, Meyers DA, Hawkins GA, Bleecker ER, Jarjour N, Cohn L, Chupp GL, Severe Asthma Research Program I (2015). Genetic variation in chitinase 3-like 1 (CHI3L1) contributes to asthma severity and airway expression of YKL-40. J Allergy Clin Immunol.

[53] Li JM, Zhang HF, Shen XL, Xie H, Wu XD, Shen T, Wang Y (2015). Association between CHI3L1 SNPs and susceptibility to childhood asthma. Zhongguo Dang Dai Er Ke Za Zhi.

[54] Hector A, Kormann MS, Mack I, Latzin P, Casaulta C, Kieninger E, Zhou Z, Yildirim AO, Bohla A, Rieber N, Kappler M, Koller B, Eber E, Eickmeier O, Zielen S, Eickelberg O, Griese M, Mall MA, Hartl D (2011). The chitinase-like protein YKL-40 modulates cystic fibrosis lung disease. PLoS One.

[55] Santos CB, Davidson J, Covar RA, Spahn JD (2014). The chitinase-like protein YKL-40 is not a useful biomarker for severe persistent asthma in children. Ann Allergy Asthma Immunol.

[56] Konradsen JR, James A, Nordlund B, Reinius LE, Soderhall C, Melen E, Wheelock AM, Lodrup Carlsen KC, Lidegran M, Verhoek M, Boot RG, Dahlen B, Dahlen SE, Hedlin G (2013). The chitinase-like protein YKL-40: a possible biomarker of inflammation and airway remodeling in severe pediatric asthma. J Allergy Clin Immunol.

[57] Sakazaki Y, Hoshino T, Takei S, Sawada M, Oda H, Takenaka S, Imaoka H, Matsunaga K, Ota T, Abe Y, Miki I, Fujimoto K, Kawayama T, Kato S, Aizawa H (2011). Overexpression of chitinase 3-like 1/YKL-40 in lung-specific IL-18-transgenic mice, smokers and COPD. PLoS One.

[58] Letuve S, Kozhich A, Arouche N, Grandsaigne M, Reed J, Dombret MC, Kiener PA, Aubier M, Coyle AJ, Pretolani M (2008). YKL-40 is elevated in patients with chronic obstructive pulmonary disease and activates alveolar macrophages. J Immunol.

[59] Otsuka K, Matsumoto H, Niimi A, Muro S, Ito I, Takeda T, Terada K, Yamaguchi M, Matsuoka H, Jinnai M, Oguma T, Nakaji H, Inoue H, Tajiri T, Iwata T, Chin K, Mishima M (2012). Sputum YKL-40 levels and pathophysiology of asthma and chronic obstructive pulmonary disease. Respiration.

[60] He CH, Lee CG, Dela Cruz CS, Lee CM, Zhou Y, Ahangari F, Ma B, Herzog EL, Rosenberg SA, Li Y, Nour AM, Parikh CR, Schmidt I, Modis Y, Cantley L, Elias JA (2013). Chitinase 3-like 1 regulates cellular and tissue responses via IL-13 receptor α2. Cell Rep.

[61] Webb DC, McKenzie AN, Foster PS (2001). Expression of the Ym2 lectin-binding protein is dependent on interleukin (IL)-4 and IL-13 signal transduction: identification of a novel allergy-associated protein. J Biol Chem.

[62] Cai Y, Kumar RK, Zhou J, Foster PS, Webb DC (2009). Ym1/2 promotes Th2 cytokine expression by inhibiting 12/15(S)-lipoxygenase: identification of a novel pathway for regulating allergic inflammation. J Immunol.

[63] Kott E, Duquesnoy P, Copin B, Legendre M, Dastot-Le Moal F, Montantin G, Jeanson L, Tamalet A, Papon JF, Siffroi JP, Rives N, Mitchell V, de Blic J, Coste A, Clement A, Escalier D, Toure A, Escudier E, Amselem S (2012). Loss-of-function mutations in LRRC6, a gene essential for proper axonemal assembly of inner and outer dynein arms, cause primary ciliary dyskinesia. Am J Hum Genet.

[64] Raidt J, Wallmeier J, Hjeij R, Onnebrink JG, Pennekamp P, Loges NT, Olbrich H, Haffner K, Dougherty GW, Omran H, Werner C (2014). Ciliary beat pattern and frequency in genetic variants of primary ciliary dyskinesia. Eur Respir J.

[65] Amsterdam A, Nissen RM, Sun Z, Swindell EC, Farrington S, Hopkins N (2004). Identification of 315 genes essential for early zebrafish development. Proc Natl Acad Sci U S A.

[66] Sun Z, Amsterdam A, Pazour GJ, Cole DG, Miller MS, Hopkins N (2004). A genetic screen in zebrafish identifies cilia genes as a principal cause of cystic kidney. Development.

[67] Kishimoto N, Cao Y, Park A, Sun Z (2008). Cystic kidney gene seahorse regulates cilia-mediated processes and Wnt pathways. Dev Cell.

[68] Zhao L, Yuan S, Cao Y, Kallakuri S, Li Y, Kishimoto N, DiBella L, Sun Z (2013). Reptin/Ruvbl2 is a Lrrc6/Seahorse interactor essential for cilia motility. Proc Natl Acad Sci U S A.

[69] Inaba Y, Shinohara K, Botilde Y, Nabeshima R, Takaoka K, Ajima R, Lamri L, Takeda H, Saga Y, Nakamura T, Hamada H (2016). Transport of the outer dynein arm complex to cilia requires a cytoplasmic protein Lrrc6. Genes Cells.

[70] Kott E, Legendre M, Copin B, Papon JF, Dastot-Le Moal F, Montantin G, Duquesnoy P, Piterboth W, Amram D, Bassinet L, Beucher J, Beydon N, Deneuville E, Houdouin V, Journel H, Just J, Nathan N, Tamalet A, Collot N, Jeanson L, Le Gouez M, Vallette B, Vojtek AM, Epaud R, Coste A, Clement A, Housset B, Louis B, Escudier E, Amselem S (2013). Loss-of-function mutations in RSPH1 cause primary ciliary dyskinesia with central-complex and radial-spoke defects. Am J Hum Genet.

[71] Knowles MR, Ostrowski LE, Leigh MW, Sears PR, Davis SD, Wolf WE, Hazucha MJ, Carson JL, Olivier KN, Sagel SD, Rosenfeld M, Ferkol TW, Dell SD, Milla CE, Randell SH, Yin W, Sannuti A, Metjian HM, Noone PG, Noone PJ, Olson CA, Patrone MV, Dang H, Lee HS, Hurd TW, Gee HY, Otto EA, Halbritter J, Kohl S, Kircher M, Krischer J, Bamshad MJ, Nickerson DA, Hildebrandt F, Shendure J, Zariwala MA (2014). Mutations in RSPH1 cause primary ciliary dyskinesia with a unique clinical and ciliary phenotype. Am J Respir Crit Care Med.

[72] Walther A, Riehemann K, Gerke V (2000). A novel ligand of the formyl peptide receptor: annexin I regulates neutrophil extravasation by interacting with the FPR. Mol Cell.

[73] Grewal T, Enrich C (2009). Annexins--modulators of EGF receptor signalling and trafficking. Cell Signal.

[74] Cardini S, Dalli J, Fineschi S, Perretti M, Lungarella G, Lucattelli M (2012). Genetic ablation of the fpr1 gene confers protection from smoking-induced lung emphysema in mice. Am J Respir Cell Mol Biol.

[75] Stockley RA, Grant RA, Llewellyn-Jones CG, Hill SL, Burnett D (1994). Neutrophil formyl-peptide receptors. Relationship to peptide-induced responses and emphysema. Am J Respir Crit Care Med.

[76] Matheson M, Rynell AC, McClean M, Berend N (2003). Cigarette smoking increases neutrophil formyl methionyl leucyl phenylalanine receptor numbers. Chest.

[77] Vacchelli E, Ma Y, Baracco EE, Sistigu A, Enot DP, Pietrocola F, Yang H, Adjemian S, Chaba K, Semeraro M, Signore M, De Ninno A, Lucarini V, Peschiaroli F, Businaro L, Gerardino A, Manic G, Ulas T, Gunther P, Schultze JL, Kepp O, Stoll G, Lefebvre C, Mulot C, Castoldi F, Rusakiewicz S, Ladoire S, Apetoh L, Bravo-San Pedro JM, Lucattelli M, Delarasse C, Boige V, Ducreux M, Delaloge S, Borg C, Andre F, Schiavoni G, Vitale I, Laurent-Puig P, Mattei F, Zitvogel L, Kroemer G (2015). Chemotherapy-induced antitumor immunity requires formyl peptide receptor 1. Science.

[78] Zhang Y, Ge C, Wang L, Liu X, Chen Y, Li M, Zhang M (2015). Induction of DKK1 by ox-LDL negatively regulates intracellular lipid accumulation in macrophages. FEBS Lett.

[79] Malur A, Huizar I, Wells G, Barna BP, Malur AG, Thomassen MJ (2011). Lentivirus-ABCG1 instillation reduces lipid accumulation and improves lung compliance in GM-CSF knock-out mice. Biochem Biophys Res Commun.

[80] Thomassen MJ, Barna BP, Malur AG, Bonfield TL, Farver CF, Malur A, Dalrymple H, Kavuru MS, Febbraio M (2007). ABCG1 is deficient in alveolar macrophages of GM-CSF knockout mice and patients with pulmonary alveolar proteinosis. J Lipid Res.

[81] Baldan A, Tarr P, Vales CS, Frank J, Shimotake TK, Hawgood S, Edwards PA (2006). Deletion of the transmembrane transporter ABCG1 results in progressive pulmonary lipidosis. J Biol Chem.

[82] Romero F, Shah D, Duong M, Penn RB, Fessler MB, Madenspacher J, Stafstrom W, Kavuru M, Lu B, Kallen CB, Walsh K, Summer R (2015). A pneumocyte-macrophage paracrine lipid axis drives the lung toward fibrosis. Am J Respir Cell Mol Biol.

[83] Baldan A, Gomes AV, Ping P, Edwards PA (2008). Loss of ABCG1 results in chronic pulmonary inflammation. J Immunol.

[84] Baldan A, Gonen A, Choung C, Que X, Marquart TJ, Hernandez I, Bjorkhem I, Ford DA, Witztum JL, Tarling EJ (2014). ABCG1 is required for pulmonary B-1 B cell and natural antibody homeostasis. J Immunol.

[85] Granada M, Wilk JB, Tuzova M, Strachan DP, Weidinger S, Albrecht E, Gieger C, Heinrich J, Himes BE, Hunninghake GM, Celedon JC, Weiss ST, Cruikshank WW, Farrer LA, Center DM, O'Connor GT (2012). A genome-wide association study of plasma total IgE concentrations in the Framingham Heart Study. J Allergy Clin Immunol.

[86] Mittal RA, Hammel M, Schwarz J, Heschl KM, Bretschneider N, Flemmer AW, Herber-Jonat S, Konigshoff M, Eickelberg O, Holzinger A (2012). SFTA2--a novel secretory peptide highly expressed in the lung--is modulated by lipopolysaccharide but not hyperoxia. PLoS One.

[87] Rausch F, Schicht M, Paulsen F, Ngueya I, Brauer L, Brandt W (2012). “SP-G”, a putative new surfactant protein--tissue localization and 3D structure. PLoS One.

[88] May-Simera HL, Kelley MW (2012). Cilia, Wnt signaling, and the cytoskeleton. Cilia.

