# Supplementary material for: Transcriptomic analysis comparing mouse strains with extreme total lung capacities identifies novel candidate genes for pulmonary function
Source: Respir Res. 2017 Aug 9;18:152. doi: 10.1186/s12931-017-0629-3 (PMC5551015; doi:10.1186/s12931-017-0629-3)
Supplement: Additional file 1: Table S1. — Increased lung transcripts in JF1/MsJ (JF1) compared to C3H/HeJ (C3H) mice at embryonic day 18. Table S2. Decreased lung transcripts in JF1/MsJ (JF1) compared to C3H/HeJ (C3H) mice at embryonic stage 18. Table S3. Increased lung transcripts in JF1/MsJ (JF1) compared to C3H/HeJ (C3H) mice at postnatal day 28. Table S4. Decreased lung transcripts in JF1/MsJ (JF1) compared to C3H/HeJ (C3H) mice at postnatal day 28. Table S5. Increased lung transcripts in JF1/MsJ (JF1) compared to C3H/HeJ (C3H) mice at postnatal day 70. Table S6. Decreased lung transcripts in JF1/MsJ (JF1) compared to C3H/HeJ (C3H) mice at postnatal day 70. Table S7. Transcripts showing consistent pattern of expression lung transcripts in JF1/MsJ (JF1) compared to C3H/HeJ (C3H) mice across E18/ P28 stages. Table S8. Transcripts showing consistent pattern of expression lung transcripts in JF1/MsJ (JF1) compared to C3H/HeJ (C3H) mice across P28/ P70 stages. Figure S1. Representative lung sections showing smaller alveoli in JF1/Msf (JF1) mice compared to C3H/HeJ (C3H) in both males and females. (DOCX 216 kb) [file 12931_2017_629_MOESM1_ESM.docx]

**Additional file 1**

**Gene expression profiling of mouse lungs with extreme total lung capacities identifies novel candidate genes for pulmonary function**

Leema George^1^*, Ankita Mitra^1^*, Tania A Thimraj^1^*, Martin Irmler^2*^, Sangeetha Vishweswaraiah^1^, Lars Lunding^3^, Dorothea Hühn^4$^, Alicia Madurga^5^, Johannes Beckers^2,6,7^, Heinz Fehrenbach^8^, Swapna Upadhyay^9,10^, Holger Schulz^11,12#^, George D Leikauf^13#^, Koustav Ganguly^1,,9,10,14#†^

| ^1^SRM Research Institute, SRM University, Chennai 603203 India |
| --- |
| ^2^Institute of Experimental Genetics, Helmholtz Zentrum Muenchen, German Research Center for  Environmental Health, Neuherberg, Munich, 85764 Germany |
| ^3^Priority Area Asthma & Allergy, Division of Asthma Exacerbation & Regulation, Research Center Borstel, Airway Research Center North (ARCN), Member of the German Center of Lung Research (DZL), 23845 Borstel, Germany |
| ^4^Department of Medicine, Pulmonary and Critical Care Medicine, University Medical Centre  Giessen and Marburg, Philipps-University Marburg, Member of the German Centre for Lung Research (DZL), Marburg, Germany |
| ^5^Department of Internal Medicine (Pulmonology), University of Giessen and Marburg Lung  Center (UGMLC), Member of the German Center of Lung Research (DZL), 35392 Giessen, Germany |
| ^6^German Center for Diabetes Research (DZD), 85764 Neuherberg, Germany |
| ^7^Technische Universität München, Chair of Experimental Genetics, 85354 Freising, Germany  ^8^Priority Area Asthma & Allergy, Division of Experimental Pneumology, Research Center Borstel,  Airway Research Center North (ARCN), Member of the German Center of Lung Research  (DZL), 23845 Borstel, Germany |
| ^9^Lung and Airway Research, Institute of Environmental Medicine, Karolinska Institutet Box 287, SE-171 77 Stockholm, Sweden |
| ^10^Institute of Lung Biology and Disease, Helmholtz Zentrum Muenchen, German Research Center for Environmental Health, Neuherberg, Munich, 85764 Germany |
| ^11^Institute of Epidemiology I, Helmholtz Zentrum Muenchen, German Research Center for  Environmental Health, Neuherberg, Munich, 85764 Germany |
| ^12^Comprehensive Pneumology Center Munich (CPC-M), Member of the German Center for Lung  Research, Munich, Germany |
| ^13^Department of Environmental and Occupational Health, Graduate School of Public Health,  University of Pittsburgh, Pittsburgh, Pennsylvania, 15219, USA |
| ^14^Work Environment Toxicology; Institute of Environmental Medicine, Karolinska Institutet; Box  287, SE-171 77 Stockholm, Sweden |
| ^*,#^ Contributed Equally; ^†^ Correspondence  ^$^Present address: Lahn-Dill-Kliniken, Klinikum Wetzlar, Medizinische Klinik II, Forsthausstraße 1, D-35578 Wetzlar  **Running title:** Lung function candidate genes in mice |

**Correspondence:** Koustav Ganguly, PhD, Units of Lung and Airway Research & Work Environment Toxicology, Institute of Environmental Medicine, Karolinska Institutet; Box 287, SE-171 77 Stockholm, Sweden **Email:** koustav.ganguly@ki.se; Phone: +46-0852487133

**Additional file 1**

**Contents:**

|  |  |  |
| --- | --- | --- |
| **Table S1**. Increased lung transcripts in JF1/MsJ (JF1) compared to C3H/HeJ (C3H) mice at embryonic day 18 |  |  |
| **Table S2**. Decreased lung transcripts in JF1/MsJ (JF1) compared to C3H/HeJ (C3H) mice at embryonic stage 18 |  |  |
| **Table S3**. Increased lung transcripts in JF1/MsJ (JF1) compared to C3H/HeJ (C3H) mice at postnatal day 28 |  |  |
| **Table S4.** Decreased lung transcripts in JF1/MsJ (JF1) compared to C3H/HeJ (C3H) mice at postnatal day 28 |  |  |
| **Table S5.** Increased lung transcripts in JF1/MsJ (JF1) compared to C3H/HeJ (C3H) mice at postnatal day 70 |  |  |
| **Table S6.** Decreased lung transcripts in JF1/MsJ (JF1) compared to C3H/HeJ (C3H) mice at postnatal day 70 |  |  |
| **Table S7.** Transcripts showing consistent pattern of expression lung transcripts in JF1/MsJ (JF1) compared to C3H/HeJ (C3H) mice across E18/ P28 stages |  |  |
| **Table S8.** Transcripts showing consistent pattern of expression lung transcripts in JF1/MsJ (JF1) compared to C3H/HeJ (C3H) mice across P28/ P70 stages |  |  |
| **Figure S1.** Representative lung sections showing smaller alveoli in JF1/Msf (JF1) mice compared to C3H/HeJ (C3H) in both males and females |  |  |

**Supplementary Tables:**

**Table S1**. Increased lung transcripts in JF1/MsJ (JF1) compared to C3H/HeJ (C3H) mice at embryonic day 18 [cut off for fold change ≥ 2 fold; false discovery rate <10%; total number of transcripts= 45]

| **Gene Name** | **Gene symbol** | **Entrez ID** | **Probeset** | **JF1/C3H** |
| --- | --- | --- | --- | --- |
| Glutaredoxin 3 | *Glrx3* | 30926 | 6923313 | 12.22 |
| Predicted gene 14406 | *Gm14406* | 100504328 | 6894013 | 4.58 |
| Troponin C2, fast | *Tnnc2* | 21925 | 6892955 | 4.03 |
| Zinc finger protein 125 | *Zfp125* | 22651 | 7006273 | 3.92 |
| RIKEN cDNA G730007D18 gene | *G730007D18Rik* | 100038502 | 7006004 | 3.66 |
| Holliday junction recognition protein | *Hjurp* | 381280 | 6760490 | 3.58 |
| Phospholipid phosphatase related 5 | *Plppr5* | 75769 | 6900768 | 3.23 |
| Ribosomal protein L28 | *Rpl28* | 19943 | 6972641 | 3.20 |
| Synaptonemal complex central element protein 1 | *Syce1* | 74075 | 6972143 | 3.00 |
| Predicted gene 14403 | *Gm14403* | 433520 | 6883977 | 2.98 |
| RIKEN cDNA 2610028J07 gene | *2610028J07Rik* | 71813 | 6859453 | 2.94 |
| Predicted gene 10827 | *Gm10827* | 100038405 | 6766281 | 2.92 |
| Nebulin | *Neb* | 17996 | 6886729 | 2.78 |
| Paternally expressed 10 | *Peg10* | 170676 | 6943841 | 2.74 |
| Predicted gene 3594 | *Gm3594* | 100041964 | 6816697 | 2.70 |
| Mex3 homolog A (C. Elegans) | *Mex3a* | 72640 | 6899140 | 2.66 |
| Microspherule protein 1 | *Mcrs1* | 51812 | 6918337 | 2.62 |
| Myosin binding protein C, slow-type | *Mybpc1* | 109272 | 6775854 | 2.60 |
| RIKEN cDNA 2610507I01 gene | *2610507I01Rik* | 72203 | 6788659 | 2.60 |
| H2A histone family, member X | *H2afx* | 15270 | 6988627 | 2.52 |
| Olfactory receptor 1061 | *Olfr1061* | 259022 | 6888451 | 2.51 |
| Olfactory receptor 170 | *Olfr170* | 258959 | 6844403 | 2.47 |
| Immunoglobulin superfamily, member 10 | *Igsf10* | 242050 | 6905432 | 2.43 |
| Fatty acyl CoA reductase 2 | *Far2* | 330450 | 6951180 | 2.42 |
| Lipopolysaccharide binding protein | *Lbp* | 16803 | 6882730 | 2.36 |
| Uncharacterized protein C130090J04 | *C130090J04* | 328049 | 6860959 | 2.36 |
| RIKEN cDNA 6330403K07 gene | *6330403K07Rik* | 103712 | 6789483 | 2.32 |
| Eosinophil-associated, ribonuclease A family, member 2 | *Ear2* | 13587 | 7003605 | 2.31 |
| Histone cluster 1, H2bk | *Hist1h2bk* | 319184 | 6805270 | 2.24 |
| Troponin T3, skeletal, fast | *Tnnt3* | 21957 | 6965269 | 2.22 |
| Predicted gene 3785 | *Gm3785* | 100042311 | 6807753 | 2.19 |
| Proline arginine-rich end leucine-rich repeat | *Prelp* | 116847 | 6762328 | 2.19 |
| Aldo-keto reductase family 1, member E1 | *Akr1e1* | 56043 | 6810717 | 2.16 |
| PDZ and LIM domain 3 | *Pdlim3* | 53318 | 6975871 | 2.15 |
| Lumican | *Lum* | 17022 | 6770072 | 2.14 |
| Processing of precursor 4, ribonuclease P/MRP family, (S. cerevisiae) | *Pop4* | 66161 | 6966611 | 2.12 |
| Coatomer protein complex, subunit zeta 2 | *Copz2* | 56358 | 6783873 | 2.11 |
| Growth arrest specific 5 | *Gas5* | 14455 | 6754519 | 2.09 |
| 3'-phosphoadenosine 5'-phosphosulfate synthase 2 | *Papss2* | 23972 | 6869216 | 2.06 |
| Inner centromere protein | *Incenp* | 16319 | 6871457 | 2.06 |
| TraB domain containing 2B | *Trabd2b* | 666048 | 6916450 | 2.05 |
| Neuropilin (NRP) and tolloid (TLL)-like 2 | *Neto2* | 74513 | 6983950 | 2.05 |
| Solute carrier family 22 (organic anion transporter), member 19 | *Slc22a19* | 207151 | 6871332 | 2.03 |
| Leukocyte cell derived chemotaxin 1 | *Lect1* | 16840 | 6826285 | 2.03 |
| Chemokine (C-C motif) ligand 11 | *Ccl11* | 20292 | 6782917 | 2.00 |

**Table S2**. Decreased lung transcripts in JF1/MsJ (JF1) compared to C3H/HeJ (C3H) mice at embryonic stage 18 [ cut off for fold change ≥ 2 fold; false discovery rate <10%;total number of transcripts= 102]

| **Gene Name** | **Genesymbol** | **Entrez ID** | **Probeset** | **JF1/C3H** |
| --- | --- | --- | --- | --- |
| Chemokine (C-X-C motif) ligand 15 | *Cxcl15* | 20309 | 6932372 | -14.60 |
| RIKEN cDNA A730017L22 gene | *A730017L22Rik* | 613258 | 6890967 | -13.13 |
| Ring finger protein 19A pseudogene | *Gm7444* | 665005 | 6989287 | -12.33 |
| Hemolytic complement | *Hc* | 15139 | 6886022 | -8.61 |
| Kallikrein 1-related peptidase b21 | *Klk1b21* | 16616 | 6960239 | -8.25 |
| Secretoglobin, family 1A, member 1 (uteroglobin) | *Scgb1a1* | 22287 | 6871425 | -7.34 |
| Zinc finger, X-linked, duplicated B | *Zxdb* | 668166 | 7021109 | -6.18 |
| G protein-coupled receptor 137B | *Gpr137b* | 83924 | 7003081 | -5.39 |
| CD36 antigen | *Cd36* | 12491 | 6936406 | -5.11 |
| RIKEN cDNA B930063I24 gene | *B930063I24Rik* | 319330 | 6756745 | -5.06 |
| Methyltransferase like 21E | *Mettl21e* | 403183 | 6758367 | -4.49 |
| RIKEN cDNA 4930503E14 gene | *4930503E14Rik* | 74954 | 7008694 | -4.32 |
| Transmembrane protein 181A | *Tmem181a* | 77106 | 6812918 | -4.26 |
| Chloride intracellular channel 5 | *Clic5* | 224796 | 6850564 | -4.07 |
| Stearoyl-Coenzyme A desaturase 1 | *Scd1* | 20249 | 6873271 | -4.07 |
| RIKEN cDNA 1300014J16 gene | *1300014J16Rik* | 74160 | 6796370 | -4.02 |
| Surfactant associated 2 | *Sfta2* | 433102 | 6850183 | -4.02 |
| A kinase (PRKA) anchor protein 5 | *Akap5* | 238276 | 6796173 | -3.84 |
| Surfactant associated protein A1 | *Sftpa1* | 20387 | 6818499 | -3.80 |
| Nuclear paraspeckle assembly transcript 1 (non-protein coding) | *Neat1* | 66961 | 6867774 | -3.54 |
| Predicted gene 5141 | *Gm5141* | 380850 | 6813793 | -3.47 |
| Lysosomal-associated membrane protein 3 | *Lamp3* | 239739 | 6844411 | -3.25 |
| C-type lectin domain family 2, member d | *Clec2d* | 93694 | 6950125 | -3.18 |
| Sodium channel, voltage-gated, type VII, alpha | *Scn7a* | 20272 | 6887380 | -3.17 |
| Collagen, type XXVIII, alpha 1 | *Col28a1* | 213945 | 6951538 | -3.15 |
| Mesothelin | *Msln* | 56047 | 6854431 | -3.00 |
| RIKEN cDNA D230046O15 gene | *D230046O15Rik* | 106824 | 6856766 | -3.00 |
| Paraoxonase 3 | *Pon3* | 269823 | 6951401 | -2.96 |
| Ceruloplasmin | *Cp* | 12870 | 6896032 | -2.95 |
| Lysozyme 2 | *Lyz2* | 17105 | 6777310 | -2.88 |
| Expressed sequence AL118220 | *AL118220* | 404583 | 6815681 | -2.83 |
| Aminolevulinate, delta-, dehydratase | *Alad* | 17025 | 6922241 | -2.82 |
| Xanthine dehydrogenase | *Xdh* | 22436 | 6857183 | -2.80 |
| SEC14-like 3 (S. cerevisiae) | *Sec14l3* | 380683 | 6778363 | -2.76 |
| Cathepsin H | *Ctsh* | 13036 | 6991264 | -2.75 |
| Endomucin | *Emcn* | 59308 | 6901671 | -2.71 |
| Indolethylamine N-methyltransferase | *Inmt* | 21743 | 6953810 | -2.66 |
| Flavin containing monooxygenase 1 | *Fmo1* | 14261 | 6763623 | -2.63 |
| Interleukin 18 receptor 1 | *Il18r1* | 16182 | 6748889 | -2.58 |
| Stefin A3 | *Stfa3* | 20863 | 6845416 | -2.53 |
| Predicted gene 9801 | *Gm9801* | 330552 | 6967803 | -2.49 |
| DNA-damage regulated autophagy modulator 1 | *Dram1* | 71712 | 6775838 | -2.48 |
| RIKEN cDNA 3110037C07 gene | *3110037C07Rik* | 73125 | 6895977 | -2.47 |
| Integrin beta 6 | *Itgb6* | 16420 | 6887088 | -2.47 |
| RIKEN cDNA 9330182L06 gene | *9330182L06Rik* | 231014 | 6928759 | -2.47 |
| Cytochrome P450, family 2, subfamily f, polypeptide 2 | *Cyp2f2* | 13107 | 6959279 | -2.43 |
| Neuron-derived neurotrophic factor | *Ndnf* | 68169 | 6946750 | -2.41 |
| Sodium channel, nonvoltage-gated 1 gamma | *Scnn1g* | 20278 | 6964011 | -2.41 |
| RIKEN cDNA B230114P17 gene | *B230114P17Rik* | 402735 | 6776760 | -2.40 |
| Lysozyme 2 | *Lyz2* | 17105 | 6777309 | -2.39 |
| Vascular cell adhesion molecule 1 | *Vcam1* | 22329 | 6908486 | -2.37 |
| Dipeptidase 1 (renal) | *Dpep1* | 13479 | 6979680 | -2.36 |
| RIKEN cDNA A330076H08 gene | *A330076H08Rik* | 320026 | 6967799 | -2.35 |
| RIKEN cDNA A230057D06 gene | *A230057D06Rik* | 319893 | 6967787 | -2.32 |
| WD repeat domain 45 | *Wdr45* | 54636 | 7009782 | -2.32 |
| Cytochrome P450, family 2, subfamily j, polypeptide 6 | *Cyp2j6* | 13110 | 6923520 | -2.29 |
| RIKEN cDNA D830024F11 gene | *D830024F11Rik* | 320911 | 6903545 | -2.26 |
| Predicted gene 14399 | *Gm14399* | 100043761 | 6893683 | -2.25 |
| UDP-glucose glycoprotein Glucosyltransferase 2 | *Uggt2* | 66435 | 6827825 | -2.25 |
| Alcohol dehydrogenase 1 (class I) | *Adh1* | 11522 | 6901737 | -2.24 |
| Megalencephalicleukoencephalopathy with subcortical cysts 1 homolog (human) | *Mlc1* | 170790 | 6837773 | -2.24 |
| Fibroblast growth factor 1 | *Fgf1* | 14164 | 6864837 | -2.21 |
| Gamma-aminobutyric acid (GABA) B receptor, 1 | *Gabbr1* | 54393 | 6850271 | -2.20 |
| Protein phosphatase 1, regulatory (inhibitor) subunit 3A | *Ppp1r3a* | 140491 | 6951783 | -2.19 |
| Family with sequence similarity 198, member B | *Fam198b* | 68659 | 6898630 | -2.18 |
| EGF-like, fibronectin type III and laminin G domains | *Egflam* | 268780 | 6833788 | -2.13 |
| NME/NM23 family member 7 | *Nme7* | 171567 | 6754701 | -2.13 |
| Potassium inwardly-rectifying channel, subfamily J, member 15 | *Kcnj15* | 16516 | 6843419 | -2.13 |
| Coiled-coil domain containing 59 | *Ccdc59* | 52713 | 6770445 | -2.13 |
| Transmembrane protein 181A | *Tmem181a* | 77106 | 6848520 | -2.13 |
| Transferrin | *Trf* | 22041 | 6998397 | -2.12 |
| Predicted gene 10563 | *Gm10563* | 100038426 | 6927270 | -2.11 |
| G protein-coupled receptor, family C, group 6, member A | *Gprc6a* | 210198 | 6773918 | -2.11 |
| Interferon-induced protein 44 | *Ifi44* | 99899 | 6910592 | -2.10 |
| Tandem C2 domains, nuclear | *Tc2n* | 74413 | 6803132 | -2.10 |
| Oncostatin M receptor | *Osmr* | 18414 | 6833736 | -2.10 |
| Gelsolin | *Gsn* | 227753 | 6876380 | -2.10 |
| LIM domain only 7 | *Lmo7* | 380928 | 6821434 | -2.09 |
| Schlafen 5 | *Slfn5* | 327978 | 6782979 | -2.09 |
| Predicted gene 12680 | *Gm12680* | 329876 | 6915071 | -2.09 |
| Tubulointerstitial nephritis antigen | *Tinag* | 26944 | 6997034 | -2.09 |
| Sterile alpha motif domain containing 9-like | *Samd9l* | 209086 | 6951281 | -2.08 |
| RIKEN cDNA 9930111J21 gene 2 | *9930111J21Rik2* | 245240 | 6787925 | -2.08 |
| Sodium channel, voltage-gated, type III, alpha | *Scn3a* | 20269 | 6887324 | -2.08 |
| RAD9 homolog B (S. cerevisiae) | *Rad9b* | 231724 | 6941734 | -2.07 |
| Solute carrier family 39 (metal ion transporter), member 8 | *Slc39a8* | 67547 | 6901634 | -2.07 |
| Transmembrane protein 139 | *Tmem139* | 109218 | 6945776 | -2.06 |
| Transmembrane protein 178 | *Tmem178* | 68027 | 6852542 | -2.06 |
| Coiled-coil domain containing 141 | *Ccdc141* | 545428 | 6888008 | -2.06 |
| Formyl peptide receptor 1 | *Fpr1* | 14293 | 6854043 | -2.05 |
| LIM domain containing preferred translocation partner in lipoma | *Lpp* | 210126 | 6840199 | -2.05 |
| Interleukin 33 | *Il33* | 77125 | 6869068 | -2.04 |
| Ros1 proto-oncogene | *Ros1* | 19886 | 6773933 | -2.03 |
| Leucine rich repeat containing 6 (testis) | *Lrrc6* | 54562 | 6836323 | -2.03 |
| Expressed sequence AA987161 | *AA987161* | 100416706 | 6814070 | -2.03 |
| Functional intergenic repeating RNA element | *Firre* | 103012 | 7016782 | -2.02 |
| Solute carrier family 24 (sodium/potassium/calcium exchanger), member 4 | *Slc24a4* | 238384 | 6797476 | -2.02 |
| Glucagon-like peptide 1 receptor | *Glp1r* | 14652 | 6849762 | -2.02 |
| RIKEN cDNA 4930444F02 gene | *4930444F02Rik* | 73968 | 6772413 | -2.02 |
| Transmembrane 4 superfamily member 1 | *Tm4sf1* | 17112 | 6905289 | -2.01 |
| Sema domain, immunoglobulin domain (Ig), short basic domain, secreted, (semaphorin) 3E | *Sema3e* | 20349 | 6928880 | -2.01 |
| Solute carrier family 36 (proton/amino acid symporter), member 2 | *Slc36a2* | 246049 | 6788401 | -2.00 |

**Table S3.** Increased lung transcripts in JF1/MsJ (JF1) compared to C3H/HeJ (C3H) mice at postnatal day 28 [cut off for fold change ≥ 2 fold; false discovery rate <10%;total number of transcripts= 65]

| **Gene Name** | **Gene symbol** | **Entrez ID** | **Probeset** | **JF1/C3H** |
| --- | --- | --- | --- | --- |
| Serine peptidase inhibitor, Kazal type 5 | *Spink5* | 72432 | 6860513 | 12.79 |
| WAP four-disulfide core domain 10 | *Wfdc10* | 629756 | 6883109 | 9.45 |
| Olfactory receptor 170 | *Olfr170* | 258959 | 6844409 | 7.88 |
| RIKEN cDNA 6330403K07 gene | *6330403K07Rik* | 103712 | 6789483 | 7.15 |
| Olfactory receptor 170 | *Olfr170* | 258959 | 6844407 | 6.81 |
| Follistatin | *Fst* | 14313 | 6816226 | 6.41 |
| Glutaredoxin 3 | *Glrx3* | 30926 | 6923313 | 6.08 |
| Wnt inhibitory factor 1 | *Wif1* | 24117 | 6771207 | 6.02 |
| RIKEN cDNA B430010I23 gene | *B430010I23Rik* | 78849 | 6975675 | 5.95 |
| Ankyrin repeat domain 63 | *Ankrd63* | 383787 | 6890143 | 5.93 |
| Arylsulfatasei | *Arsi* | 545260 | 6861349 | 5.71 |
| RIKEN cDNA 2610507I01 gene | *2610507I01Rik* | 72203 | 6788659 | 5.35 |
| Olfactory receptor 170 | *Olfr170* | 258959 | 6844403 | 4.67 |
| Pyrin domain containing 4 | *Pydc4* | 623121 | 6764233 | 4.22 |
| Vomeronasal 2, receptor 8 | *Vmn2r8* | 627479 | 6940964 | 4.01 |
| RIKEN cDNA 1700093K21 gene | *1700093K21Rik* | 67358 | 6786660 | 3.12 |
| Predicted gene 14406 | *Gm14406* | 100504328 | 6894013 | 3.07 |
| Cytochrome P450, family 2, subfamily a, polypeptide 4de 5 | *Cyp2a4* | 13086 | 6959265 | 2.98 |
| Tripartite motif-containing 12A | *Trim12a* | 76681 | 6970053 | 2.96 |
| Asporin | *Aspn* | 66695 | 6806958 | 2.87 |
| RIKEN cDNA G730007D18 gene | *G730007D18Rik* | 100038502 | 7006004 | 2.87 |
| RIKEN cDNA E230023K05 gene | *E230023K05Rik* | 319435 | 6828485 | 2.82 |
| Fetuin beta | *Fetub* | 59083 | 6840094 | 2.82 |
| Olfactory receptor 1061 | *Olfr1061* | 259022 | 6888451 | 2.81 |
| Radial spoke head 1 homolog (Chlamydomonas) | *Rsph1* | 22092 | 6854792 | 2.81 |
| Predicted gene 14403 | *Gm14403* | 433520 | 6883977 | 2.79 |
| Proteoglycan 4 (megakaryocyte stimulating factor, articular superficial zone protein) | *Prg4* | 96875 | 6762967 | 2.77 |
| RIKEN cDNA 2610028J07 gene | *2610028J07Rik* | 71813 | 6859453 | 2.74 |
| Pyrin domain containing 3 | *Pydc3* | 100033459 | 6755288 | 2.66 |
| Cytochrome P450, family 3, subfamily a, polypeptide 13 | *Cyp3a13* | 13113 | 6942587 | 2.65 |
| Predicted gene 2115 | *Gm2115* | 100039239 | 6962238 | 2.47 |
| Uroplakin 1B | *Upk1b* | 22268 | 6845587 | 2.44 |
| Mitogen-activated protein kinase kinasekinase 6 | *Map3k6* | 53608 | 6917577 | 2.34 |
| Expressed sequence AU021092 | *AU021092* | 239691 | 6843691 | 2.33 |
| Chordin-like 1 | *Chrdl1* | 83453 | 7019944 | 2.33 |
| Rho GTPase activating protein 20 | *Arhgap20* | 244867 | 6989086 | 2.29 |
| Lipopolysaccharide binding protein | *Lbp* | 16803 | 6882730 | 2.28 |
| RIKEN cDNA 2610028H24 gene | *2610028H24Rik* | 76964 | 6768925 | 2.27 |
| Acyl-CoA synthetase medium-chain family member 1 | *Acsm1* | 117147 | 6963886 | 2.26 |
| CD200 antigen | *Cd200* | 17470 | 6845978 | 2.25 |
| Family with sequence similarity 55, member D | *Fam55d* | 244853 | 6988898 | 2.22 |
| Histocompatibility 2, D region locus 1 | *H2-D1* | 14964 | 6850135 | 2.21 |
| Chondroitin sulfate synthase 3 | *Chsy3* | 78923 | 6861281 | 2.20 |
| Lymphatic vessel endothelial hyaluronan receptor 1 | *Lyve1* | 114332 | 6970445 | 2.20 |
| A disintegrin-like and metallopeptidase (reprolysin type) with thrombospondin type 1 motif, 15 | *Adamts15* | 235130 | 6994343 | 2.15 |
| RIKEN cDNA 1700008A23 gene | *1700008A23Rik* | 75463 | 6991781 | 2.13 |
| Kinesin family member 1A | *Kif1a* | 16560 | 6760754 | 2.12 |
| Uncharacterized protein C130090J04 | *C130090J04* | 328049 | 6860959 | 2.12 |
| Holliday junction recognition protein | *Hjurp* | 381280 | 6760490 | 2.09 |
| Trans-2,3-enoyl-CoA reductase-like | *Tecrl* | 243078 | 6939590 | 2.09 |
| Cancer susceptibility candidate 4 | *Casc4* | 319996 | 6880658 | 2.08 |
| Glutathione S-transferase, mu 7 | *Gstm7* | 68312 | 6908073 | 2.08 |
| Eph receptor A7 | *Epha7* | 13841 | 6912371 | 2.07 |
| Synaptonemal complex central element protein 1 | *Syce1* | 74075 | 6972143 | 2.06 |
| Predicted gene 10544 | *Gm10544* | 100038509 | 6860152 | 2.06 |
| NEL-like 1 (chicken) | *Nell1* | 338352 | 6960628 | 2.04 |
| Doublecortin domain containing 2b | *Dcdc2b* | 100504491 | 6925585 | 2.04 |
| Ventricular zone expressed PH domain homolog 1 (zebrafish) | *Veph1* | 72789 | 6905660 | 2.04 |
| Aldo-keto reductase family 1, member E1 | *Akr1e1* | 56043 | 6810717 | 2.03 |
| Microspherule protein 1 | *Mcrs1* | 51812 | 6918337 | 2.03 |
| Secretoglobin, family 1C, member 1 | *Scgb1c1* | 338417 | 6965125 | 2.02 |
| Solute carrier family 1 (glutamate/neutral amino acid transporter), member 4 | *Slc1a4* | 55963 | 6786473 | 2.01 |
| Arachidonate 15-lipoxygenase | *Alox15* | 11687 | 6789411 | 2.01 |
| Prostate stem cell antigen | *Psca* | 72373 | 6831527 | 2.01 |
| Solute carrier family 22 (organic anion transporter), member 19 | *Slc22a19* | 207151 | 6871332 | 2.01 |

**Table S4**. Decreased lung transcripts in JF1/MsJ (JF1) compared to C3H/HeJ (C3H) mice at postnatal day 28 [cut off for fold change ≥ 2 fold; false discovery rate <10%;Total number of transcripts= 109]

| **Gene Name** | **Gene symbol** | **Entrez ID** | **Probeset** | **JF1/C3H** |
| --- | --- | --- | --- | --- |
| Hemolytic complement | *Hc* | 15139 | 6886022 | -32.16 |
| Kallikrein 1-related peptidase b21 | *Klk1b21* | 16616 | 6960239 | -19.87 |
| RIKEN cDNA A730017L22 gene | *A730017L22Rik* | 613258 | 6890967 | -15.62 |
| Chitinase 3-like 3 | *Chi3l3* | 12655 | 6907945 | -15.62 |
| Resistin like alpha | *Retnla* | 57262 | 6841362 | -11.56 |
| Uncharacterized LOC624295 | *LOC624295* | 624295 | 6792817 | -10.82 |
| Zinc finger, X-linked, duplicated B | *Zxdb* | 668166 | 7021109 | -7.68 |
| Predicted gene 4956 | *Gm4956* | 241041 | 6757322 | -7.19 |
| G protein-coupled receptor 137B | *Gpr137b* | 83924 | 7003081 | -5.38 |
| Sodium channel, voltage-gated, type III, alpha | *Scn3a* | 20269 | 6887324 | -5.31 |
| Regenerating islet-derived 3 gamma | *Reg3g* | 19695 | 6954810 | -5.17 |
| C-type lectin domain family 4, member n | *Clec4n* | 56620 | 6949744 | -4.88 |
| BAI1-associated protein 2-like 1 | *Baiap2l1* | 66898 | 6942960 | -4.53 |
| Immunoglobulin kappa chain variable 4-91 | *Igkv4-91* | 434033 | 6954474 | -4.38 |
| RIKEN cDNA B930063I24 gene | *B930063I24Rik* | 319330 | 6756745 | -4.33 |
| Histocompatibility 2, class II antigen E alpha, pseudogene | *H2-Ea-ps* | 100504404 | 6855022 | -4.29 |
| Predicted gene 10673 | *Gm10673* | 100038559 | 6907955 | -4.22 |
| BPI fold containing family A, member 1 | *Bpifa1* | 18843 | 6882437 | -4.16 |
| Transmembrane protein 181A | *Tmem181a* | 77106 | 6812918 | -4.08 |
| Leukocyte immunoglobulin-like receptor, subfamily B, member 4 | *Lilrb4* | 14728 | 6767782 | -3.80 |
| Chitinase 3-like 1 | *Chi3l1* | 12654 | 6753247 | -3.67 |
| RIKEN cDNA D230046O15 gene | *D230046O15Rik* | 106824 | 6856766 | -3.63 |
| Stathmin-like 2 | *Stmn2* | 20257 | 6895589 | -3.62 |
| Sulfotransferase family 1D, member 1 | *Sult1d1* | 53315 | 6939723 | -3.52 |
| Nuclear paraspeckle assembly transcript 1 (non-protein coding) | *Neat1* | 66961 | 6867774 | -3.45 |
| Sciellin | *Scel* | 64929 | 6821514 | -3.35 |
| Adipocyte-related X-chromosome expressed sequence 2 | *Arxes2* | 76976 | 7013981 | -3.33 |
| Chitinase, acidic | *Chia* | 81600 | 6900239 | -3.33 |
| ATPase, H+ transporting, lysosomal V0 subunit D2 | *Atp6v0d2* | 242341 | 6920056 | -3.27 |
| Chemokine (C-C motif) ligand 6 | *Ccl6* | 20305 | 6790290 | -3.20 |
| Histocompatibility 2, Q region locus 4 | *H2-Q4* | 15015 | 6850155 | -3.19 |
| Lymphocyte antigen 6 complex, locus A | *Ly6a* | 110454 | 6836728 | -3.18 |
| ATP-binding cassette, sub-family A (ABC1), member 8a | *Abca8a* | 217258 | 6792114 | -3.16 |
| Ring finger protein 19A pseudogene | *Gm7444* | 665005 | 6989287 | -3.13 |
| Guanylate binding protein 1 | *Gbp1* | 14468 | 6901952 | -3.11 |
| Cathepsin S | *Ctss* | 13040 | 6899683 | -3.07 |
| RIKEN cDNA 5730424H11 gene | *5730424H11Rik* | 100038701 | 6832562 | -3.06 |
| RIKEN cDNA 1300014J16 gene | *1300014J16Rik* | 74160 | 6796370 | -2.88 |
| Interferon-induced protein 44 | *Ifi44* | 99899 | 6910592 | -2.86 |
| Myxovirus (influenza virus) resistance 2 | *Mx2* | 17858 | 6843550 | -2.79 |
| Megalencephalicleukoencephalopathy with subcortical cysts 1 homolog (human) | *Mlc1* | 170790 | 6837773 | -2.78 |
| Macrophage expressed gene 1 | *Mpeg1* | 17476 | 6868171 | -2.74 |
| Sterile alpha motif domain containing 9-like | *Samd9l* | 209086 | 6951281 | -2.74 |
| cDNA sequence BC064078 | *BC064078* | 408064 | 6950112 | -2.67 |
| Transmembrane protein 181A | *Tmem181a* | 77106 | 6848520 | -2.60 |
| Leukocyte immunoglobulin-like receptor, subfamily A (with TM domain), member 5 | *Lilra5* | 232801 | 6972927 | -2.57 |
| Macrophage galactose N-acetyl-galactosamine specific lectin 2 | *Mgl2* | 216864 | 6782102 | -2.56 |
| Solute carrier family 16 (monocarboxylic acid transporters), member 7 | *Slc16a7* | 20503 | 6771438 | -2.55 |
| CD209a antigen | *Cd209a* | 170786 | 6980091 | -2.54 |
| Aminolevulinate, delta-, dehydratase | *Alad* | 17025 | 6922241 | -2.54 |
| RIKEN cDNA A330076H08 gene | *A330076H08Rik* | 320026 | 6967799 | -2.53 |
| NME/NM23 family member 7 | *Nme7* | 171567 | 6754701 | -2.49 |
| RIKEN cDNA 1700012D16 gene | *1700012D16Rik* | 75489 | 6910673 | -2.48 |
| CD68 antigen | *Cd68* | 12514 | 6789325 | -2.47 |
| ST6 (alpha-N-acetyl-neuraminyl-2,3-beta-galactosyl-1,3)-N-acetylgalactosaminide alpha-2,6-sialyltransferase 2 | *St6galnac2* | 20446 | 6792544 | -2.47 |
| Matrix metallopeptidase 3 | *Mmp3* | 17392 | 6986725 | -2.45 |
| Solute carrier family 39 (metal ion transporter), member 8 | *Slc39a8* | 67547 | 6901634 | -2.43 |
| Starch binding domain 1 | *Stbd1* | 52331 | 6932509 | -2.43 |
| Bone morphogenetic protein 3 | *Bmp3* | 319746 | 6932719 | -2.43 |
| High mobility group AT-hook 2, pseudogene 1 | *Hmga2-ps1* | 15365 | 6764405 | -2.42 |
| Coiled-coil domain containing 190 | *Ccdc190* | 78465 | 6755059 | -2.41 |
| Deoxyribonuclease 1-like 3 | *Dnase1l3* | 13421 | 6822443 | -2.38 |
| Scm-like with four mbt domains 2 | *Sfmbt2* | 353282 | 6874954 | -2.36 |
| MicroRNA 344 | *Mir344* | 723931 | 6967797 | -2.36 |
| Integrin alpha X | *Itgax* | 16411 | 6964382 | -2.35 |
| RIKEN cDNA 2900001G08 gene | *2900001G08Rik* | 76950 | 6849764 | -2.33 |
| Glucagon-like peptide 1 receptor | *Glp1r* | 14652 | 6849762 | -2.33 |
| CD74 antigen (invariant polypeptide of major histocompatibility complex, class II antigen-associated) | *Cd74* | 16149 | 6861341 | -2.32 |
| Cathepsin K | *Ctsk* | 13038 | 6899682 | -2.31 |
| Protein kinase, cGMP-dependent, type II | *Prkg2* | 19092 | 6940300 | -2.31 |
| Ankyrin repeat domain 1 (cardiac muscle) | *Ankrd1* | 107765 | 6872834 | -2.28 |
| Cytochrome P450, family 2, subfamily j, polypeptide 6 | *Cyp2j6* | 13110 | 6923520 | -2.27 |
| C-type lectin domain family 9, member a | *Clec9a* | 232414 | 6950148 | -2.26 |
| Ppp1r14c pseudogene | *Gm14057* | 100043766 | 6890955 | -2.26 |
| Predicted gene 10563 | *Gm10563* | 100038426 | 6927270 | -2.25 |
| Chemokine (C-X-C motif) ligand 15 | *Cxcl15* | 20309 | 6932372 | -2.25 |
| Histocompatibility 2, class II antigen E beta | *H2-Eb1* | 14969 | 6849968 | -2.23 |
| Predicted gene 11783 | *Gm11783* | 621549 | 6911321 | -2.22 |
| Transmembrane protein 2 | *Tmem2* | 83921 | 6868650 | -2.21 |
| Gap junction protein, delta 3 | *Gjd3* | 353155 | 6791302 | -2.20 |
| Peptidylprolyl isomerase C | *Ppic* | 19038 | 6865551 | -2.19 |
| Surfactant associated 2 | *Sfta2* | 433102 | 6850183 | -2.19 |
| RIKEN cDNA 5830428M24 gene | *5830428M24Rik* | 76062 | 6801480 | -2.18 |
| RIKEN cDNA 1700015C17 gene | *1700015C17Rik* | 76928 | 6910674 | -2.17 |
| Olfactory receptor 1372, pseudogene 1 | *Olfr1372-ps1* | 257871 | 6788160 | -2.17 |
| Cryptochrome 2 (photolyase-like) | *Cry2* | 12953 | 6888835 | -2.15 |
| Fatty acid binding protein 1, liver | *Fabp1* | 14080 | 6946900 | -2.14 |
| Solute carrier family 13 (sodium/sulfatesymporters), member 4 | *Slc13a4* | 243755 | 6952679 | -2.13 |
| Spermatogenesis associated glutamate (E)-rich protein 4a pseudogene | *Gm8579* | 667335 | 6958650 | -2.13 |
| Protein phosphatase 1, regulatory (inhibitor) subunit 3A | *Ppp1r3a* | 140491 | 6951783 | -2.10 |
| Lysosomal-associated protein transmembrane 5 | *Laptm5* | 16792 | 6917393 | -2.10 |
| Sema domain, immunoglobulin domain (Ig), and GPI membrane anchor, (semaphorin) 7A | *Sema7a* | 20361 | 6989438 | -2.10 |
| Placenta expressed transcript 1 | *Plet1* | 76509 | 6989010 | -2.09 |
| N-acylethanolamine acid amidase | *Naaa* | 67111 | 6939985 | -2.08 |
| RIKEN cDNA C130026I21 gene | *C130026I21Rik* | 620078 | 7003214 | -2.08 |
| Leucine rich repeat containing 33 | *Lrrc33* | 224109 | 6845154 | -2.08 |
| Glycine amidinotransferase (L-arginine:glycineamidinotransferase) | *Gatm* | 67092 | 6890448 | -2.08 |
| 2'-5' oligoadenylatesynthetase 2 | *Oas2* | 246728 | 6941647 | -2.07 |
| Sorting nexin 10 | *Snx10* | 71982 | 6946204 | -2.07 |
| TRAF-interacting protein with forkhead-associated domain, family member B | *Tifab* | 212937 | 6813474 | -2.05 |
| KLRAQ motif containing 1 | *Klraq1* | 73825 | 6852929 | -2.04 |
| Expressed sequence AL118220 | *AL118220* | 404583 | 6815681 | -2.04 |
| RIKEN cDNA 2900072G19 gene | *2900072G19Rik* | 73017 | 6832723 | -2.04 |
| KH and NYN domain containing | *Khnyn* | 219094 | 6824850 | -2.03 |
| Kelch-like 6 (Drosophila) | *Klhl6* | 239743 | 6844426 | -2.03 |
| Predicted gene 13710 | *Gm13710* | 672763 | 6888302 | -2.02 |
| RIKEN cDNA A530099J19 gene | *A530099J19Rik* | 319293 | 6811368 | -2.02 |
| Alcohol dehydrogenase 1 (class I) | *Adh1* | 11522 | 6901737 | -2.00 |
| Schlafen 4 | *Slfn4* | 20558 | 6783005 | -2.00 |

**Table S5**. Increased lung transcripts in JF1/MsJ (JF1) compared to C3H/HeJ (C3H) mice at postnatal day 70 [cut off for fold change ≥ 2 fold; false discovery rate <10%; total number of transcripts= 46]

| **Gene Name** | **Gene symbol** | **Entrez ID** | **Probeset** | **JF1/C3H** |
| --- | --- | --- | --- | --- |
| Serine peptidase inhibitor, Kazal type 5 | *Spink5* | 72432 | 6860513 | 9.13 |
| RIKEN cDNA 6330403K07 gene | *6330403K07Rik* | 103712 | 6789483 | 6.56 |
| RIKEN cDNA B430010I23 gene | *B430010I23Rik* | 78849 | 6975675 | 5.82 |
| Olfactory receptor 170 | *Olfr170* | 258959 | 6844409 | 5.47 |
| Wnt inhibitory factor 1 | *Wif1* | 24117 | 6771207 | 5.42 |
| Predicted gene 1337 | *Gm1337* | 383787 | 6890143 | 5.01 |
| Follistatin | *Fst* | 14313 | 6816226 | 4.57 |
| Olfactory receptor 170 | *Olfr170* | 258959 | 6844407 | 4.24 |
| Arylsulfatasei | *Arsi* | 545260 | 6861349 | 4.17 |
| Predicted gene 3594 | *Gm3594* | 100041964 | 6816697 | 3.87 |
| Olfactory receptor 1061 | *Olfr1061* | 259022 | 6888451 | 3.77 |
| RIKEN cDNA 2610507I01 gene | *2610507I01Rik* | 72203 | 6788659 | 3.74 |
| WAP four-disulfide core domain 10 | *Wfdc10* | 629756 | 6883109 | 3.48 |
| Olfactory receptor 170 | *Olfr170* | 258959 | 6844403 | 3.43 |
| RIKEN cDNA 2310061B05 gene | *2310061B05Rik* | 70203 | 6953974 | 3.37 |
| Glutaredoxin 3 | *Glrx3* | 30926 | 6923313 | 3.27 |
| RIKEN cDNA 2610028J07 gene | *2610028J07Rik* | 71813 | 6859453 | 2.82 |
| Predicted gene 14403 | *Gm14403* | 433520 | 6883977 | 2.68 |
| RIKEN cDNA E230023K05 gene | *E230023K05Rik* | 319435 | 6828485 | 2.63 |
| Radial spoke head 1 homolog (Chlamydomonas) | *Rsph1* | 22092 | 6854792 | 2.63 |
| RIKEN cDNA 1700093K21 gene | *1700093K21Rik* | 67358 | 6786660 | 2.62 |
| Ventricular zone expressed PH domain homolog 1 (zebrafish) | *Veph1* | 72789 | 6905660 | 2.49 |
| RIKEN cDNA G730007D18 gene | *G730007D18Rik* | 100038502 | 7006004 | 2.41 |
| Predicted gene 10687 | *Gm10687* | 100038560 | 6995050 | 2.33 |
| Microspherule protein 1 | *Mcrs1* | 51812 | 6918337 | 2.30 |
| Acyl-CoA synthetase medium-chain family member 1 | *Acsm1* | 117147 | 6963886 | 2.28 |
| RIKEN cDNA 2810416G20 gene | *2810416G20Rik* | 100040353 | 6896119 | 2.27 |
| Rho GTPase activating protein 20 | *Arhgap20* | 244867 | 6989086 | 2.21 |
| Uncharacterized protein C130090J04 | *C130090J04* | 328049 | 6860959 | 2.20 |
| Holliday junction recognition protein | *Hjurp* | 381280 | 6760490 | 2.20 |
| Cathepsin E | *Ctse* | 13034 | 6753067 | 2.14 |
| Synaptonemal complex central element protein 1 | *Syce1* | 74075 | 6972143 | 2.13 |
| Predicted gene 14322 | *Gm14322* | 626802 | 6883981 | 2.13 |
| Histocompatibility 2, D region locus 1 | *H2-D1* | 14964 | 6850135 | 2.11 |
| Aminolevulinic acid synthase 2, erythroid | *Alas2* | 11656 | 7014503 | 2.10 |
| Solute carrier family 1 (glutamate/neutral amino acid transporter), member 4 | *Slc1a4* | 55963 | 6786473 | 2.09 |
| Defensin beta 11 | *Defb11* | 246081 | 6980933 | 2.09 |
| RIKEN cDNA 4933411E06 gene | *4933411E06Rik* | 71181 | 6749325 | 2.08 |
| Aryl hydrocarbon receptor nuclear translocator-like | *Arntl* | 11865 | 6963558 | 2.07 |
| Chemokine-like factor | *Cklf* | 75458 | 6978732 | 2.06 |
| Armadillo repeat containing 3 | *Armc3* | 70882 | 6875413 | 2.05 |
| Predicted gene 2115 | *Gm2115* | 100039239 | 6962238 | 2.05 |
| Doublecortin domain containing 2b | *Dcdc2b* | 100504491 | 6925585 | 2.04 |
| Melanocortin 5 receptor | *Mc5r* | 17203 | 6861776 | 2.04 |
| RIKEN cDNA C430049B03 gene | *C430049B03Rik* | 72575 | 7016878 | 2.03 |
| Cancer susceptibility candidate 4 | *Casc4* | 319996 | 6880658 | 2.00 |

**Table S6**.Decreased lung transcripts in JF1/MsJ (JF1) compared to C3H/HeJ (C3H) mice at postnatal day 70 [cut off for fold change ≥ 2 fold; false discovery rate <10%; total number of transcripts= 127]

| **Gene Name** | **Gene symbol** | **Entrez ID** | **Probeset** | **JF1/C3H** |
| --- | --- | --- | --- | --- |
| Hemolytic complement | *Hc* | 15139 | 6886022 | -27.88 |
| Immunoglobulin kappa chain variable 4-91 | *Igkv4-91* | 434033 | 6954474 | -16.44 |
| Chitinase 3-like 3 | *Chi3l3* | 12655 | 6907945 | -15.93 |
| RIKEN cDNA A730017L22 gene | *A730017L22Rik* | 613258 | 6890967 | -14.60 |
| Kallikrein 1-related peptidase b21 | *Klk1b21* | 16616 | 6960239 | -11.92 |
| Zinc finger, X-linked, duplicated B | *Zxdb* | 668166 | 7021109 | -7.23 |
| Uncharacterized LOC624295 | *LOC624295* | 624295 | 6792817 | -7.19 |
| Sciellin | *Scel* | 64929 | 6821514 | -7.16 |
| RIKEN cDNA B930063I24 gene | *B930063I24Rik* | 319330 | 6756745 | -6.19 |
| Sulfotransferase family 1D, member 1 | *Sult1d1* | 53315 | 6939723 | -6.05 |
| BAI1-associated protein 2-like 1 | *Baiap2l1* | 66898 | 6942960 | -5.92 |
| Cryptochrome 2 (photolyase-like) | *Cry2* | 12953 | 6888835 | -5.41 |
| ATP-binding cassette, sub-family A (ABC1), member 8a | *Abca8a* | 217258 | 6792114 | -5.38 |
| C-type lectin domain family 4, member n | *Clec4n* | 56620 | 6949744 | -5.15 |
| Hepcidin antimicrobial peptide | *Hamp* | 84506 | 6966327 | -4.87 |
| Sodium channel, voltage-gated, type III, alpha | *Scn3a* | 20269 | 6887324 | -4.49 |
| Predicted gene 4956 | *Gm4956* | 241041 | 6757322 | -4.40 |
| D site albumin promoter binding protein | *Dbp* | 13170 | 6960404 | -4.36 |
| Histocompatibility 2, class II antigen E alpha, pseudogene | *H2-Ea-ps* | 100504404 | 6855022 | -4.35 |
| RIKEN cDNA 5730424H11 gene | *5730424H11Rik* | 100038701 | 6832562 | -4.29 |
| Chemokine (C-C motif) ligand 6 | *Ccl6* | 20305 | 6790290 | -3.92 |
| Cathepsin S | *Ctss* | 13040 | 6899683 | -3.73 |
| Leukocyte immunoglobulin-like receptor, subfamily B, member 4 | *Lilrb4* | 14728 | 6767782 | -3.64 |
| ATPase, H+ transporting, lysosomal V0 subunit D2 | *Atp6v0d2* | 242341 | 6920056 | -3.64 |
| Leukocyte immunoglobulin-like receptor, subfamily A (with TM domain), member 5 | *Lilra5* | 232801 | 6972927 | -3.63 |
| Matrix metallopeptidase 3 | *Mmp3* | 17392 | 6986725 | -3.62 |
| Guanylate binding protein 1 | *Gbp1* | 14468 | 6901952 | -3.47 |
| Camello-like 3 | *Cml3* | 93674 | 7002589 | -3.44 |
| Transmembrane protein 181A | *Tmem181a* | 77106 | 6812918 | -3.40 |
| Stathmin-like 2 | *Stmn2* | 20257 | 6895589 | -3.37 |
| Keratin 79 | *Krt79* | 223917 | 6838694 | -3.31 |
| Megalencephalicleukoencephalopathy with subcortical cysts 1 homolog (human) | *Mlc1* | 170790 | 6837773 | -3.13 |
| Mannose receptor, C type 1 | *Mrc1* | 17533 | 6875181 | -3.12 |
| Predicted gene 16498 | *Gm16498* | 791390 | 6840932 | -3.09 |
| Ig heavy chain V region 3-6-like | *LOC634749* | 634749 | 6803956 | -3.07 |
| G protein-coupled receptor 137B | *Gpr137b* | 83924 | 7003081 | -3.06 |
| Protein phosphatase 1, regulatory (inhibitor) subunit 3A | *Ppp1r3a* | 140491 | 6951783 | -3.06 |
| Sterile alpha motif domain containing 9-like | *Samd9l* | 209086 | 6951281 | -3.05 |
| Regulator of G-protein signaling 18 | *Rgs18* | 64214 | 6762804 | -2.89 |
| Histocompatibility 2, Q region locus 4 | *H2-Q4* | 15015 | 6850155 | -2.86 |
| Basic helix-loop-helix family, member e41 | *Bhlhe41* | 79362 | 6958256 | -2.85 |
| Insulin-like growth factor binding protein 6 | *Igfbp6* | 16012 | 6833393 | -2.83 |
| Calmodulin-like 3 | *Calml3* | 70405 | 6810682 | -2.80 |
| Cytochrome P450, family 2, subfamily e, polypeptide 1 | *Cyp2e1* | 13106 | 6965115 | -2.79 |
| Collagen, type XXVIII, alpha 1 | *Col28a1* | 213945 | 6951538 | -2.76 |
| Angiopoietin-like 7 | *Angptl7* | 654812 | 6926912 | -2.72 |
| Macrophage expressed gene 1 | *Mpeg1* | 17476 | 6868171 | -2.71 |
| NME/NM23 family member 7 | *Nme7* | 171567 | 6754701 | -2.70 |
| Nuclear paraspeckle assembly transcript 1 (non-protein coding) | *Neat1* | 66961 | 6867774 | -2.70 |
| Coiled-coil domain containing 190 | *Ccdc190* | 78465 | 6755059 | -2.69 |
| RIKEN cDNA 5330406M23 gene | *5330406M23Rik* | 76671 | 6945146 | -2.68 |
| Cytochrome b-245, beta polypeptide | *Cybb* | 13058 | 7015521 | -2.68 |
| X-linked lymphocyte-regulated 5A | *Xlr5a* | 574438 | 7017573 | -2.67 |
| Aminolevulinate, delta-, dehydratase | *Alad* | 17025 | 6922241 | -2.67 |
| C-type lectin domain family 2, member d | *Clec2d* | 93694 | 6950125 | -2.67 |
| Fatty acid binding protein 1, liver | *Fabp1* | 14080 | 6946900 | -2.67 |
| Ankyrin repeat domain 1 (cardiac muscle) | *Ankrd1* | 107765 | 6872834 | -2.66 |
| Integrin alpha X | *Itgax* | 16411 | 6964382 | -2.66 |
| Period homolog 3 (Drosophila) | *Per3* | 18628 | 6927085 | -2.64 |
| CD68 antigen | *Cd68* | 12514 | 6789325 | -2.63 |
| RIKEN cDNA 4933431K14 gene | *4933431K14Rik* | 71295 | 6991020 | -2.57 |
| Transmembrane protein 2 | *Tmem2* | 83921 | 6868650 | -2.55 |
| Complement component 1, s subcomponent | *C1s* | 50908 | 6957111 | -2.55 |
| Cholesterol 25-hydroxylase | *Ch25h* | 12642 | 6872781 | -2.54 |
| RIKEN cDNA D230046O15 gene | *D230046O15Rik* | 106824 | 6856766 | -2.54 |
| ST6 (alpha-N-acetyl-neuraminyl-2,3-beta-galactosyl-1,3)-N-acetylgalactosaminide alpha-2,6-sialyltransferase 2 | *St6galnac2* | 20446 | 6792544 | -2.52 |
| Solute carrier family 39 (metal ion transporter), member 8 | *Slc39a8* | 67547 | 6901634 | -2.51 |
| C-type lectin domain family 9, member a | *Clec9a* | 232414 | 6950148 | -2.48 |
| ATP-binding cassette, sub-family G (WHITE), member 1 | *Abcg1* | 11307 | 6849766 | -2.44 |
| Prostaglandin reductase 1 | *Ptgr1* | 67103 | 6922026 | -2.41 |
| Transmembrane protein 181A | *Tmem181a* | 77106 | 6848520 | -2.40 |
| Cytochrome P450, family 2, subfamily j, polypeptide 6 | *Cyp2j6* | 13110 | 6923520 | -2.39 |
| Predicted gene 10673 | *Gm10673* | 100038559 | 6907955 | -2.33 |
| Bone morphogenetic protein 3 | *Bmp3* | 319746 | 6932719 | -2.33 |
| CD209a antigen | *Cd209a* | 170786 | 6980091 | -2.30 |
| Protein kinase, cGMP-dependent, type II | *Prkg2* | 19092 | 6940300 | -2.29 |
| Cathepsin K | *Ctsk* | 13038 | 6899682 | -2.28 |
| Leptin receptor | *Lepr* | 16847 | 6915847 | -2.28 |
| Myxovirus (influenza virus) resistance 2 | *Mx2* | 17858 | 6843550 | -2.26 |
| Formyl peptide receptor 1 | *Fpr1* | 14293 | 6854043 | -2.26 |
| Chitinase, acidic | *Chia* | 81600 | 6900239 | -2.26 |
| Nuclear receptor subfamily 1, group D, member 2 | *Nr1d2* | 353187 | 6822946 | -2.25 |
| RIKEN cDNA A530099J19 gene | *A530099J19Rik* | 319293 | 6811368 | -2.24 |
| Surfactant associated 2 | *Sfta2* | 433102 | 6850183 | -2.23 |
| NLR family, apoptosis inhibitory protein 5 | *Naip5* | 17951 | 6815523 | -2.21 |
| Claudin 1 | *Cldn1* | 12737 | 6844819 | -2.21 |
| Oxidized low density lipoprotein (lectin-like) receptor 1 | *Olr1* | 108078 | 6957412 | -2.21 |
| Histocompatibility 2, class II antigen E beta | *H2-Eb1* | 14969 | 6849968 | -2.21 |
| cDNA sequence BC147527 | *BC147527* | 625360 | 7006836 | -2.20 |
| 2'-5' oligoadenylatesynthetase 1A | *Oas1a* | 246730 | 6941657 | -2.20 |
| cDNA sequence BC064078 | *BC064078* | 408064 | 6950112 | -2.20 |
| CD74 antigen (invariant polypeptide of major histocompatibility complex, class II antigen-associated) | *Cd74* | 16149 | 6861341 | -2.20 |
| Interferon-induced protein 44 | *Ifi44* | 99899 | 6910592 | -2.19 |
| Pre-B lymphocyte gene 3 | *Vpreb3* | 22364 | 6768898 | -2.19 |
| Paraoxonase 3 | *Pon3* | 269823 | 6951401 | -2.18 |
| Coagulation factor VII | *F7* | 14068 | 6974126 | -2.18 |
| KLRAQ motif containing 1 | *Klraq1* | 73825 | 6852929 | -2.18 |
| TYRO protein tyrosine kinase binding protein | *Tyrobp* | 22177 | 6959584 | -2.16 |
| Regulator of calcineurin 2 | *Rcan2* | 53901 | 6850540 | -2.16 |
| Placenta expressed transcript 1 | *Plet1* | 76509 | 6989010 | -2.16 |
| C-type lectin domain family 7, member a | *Clec7a* | 56644 | 6957410 | -2.14 |
| RIKEN cDNA 9930111J21 gene 2 | *9930111J21Rik2* | 245240 | 6787925 | -2.13 |
| EGF-like module containing, mucin-like, hormone receptor-like sequence 1 | *Emr1* | 13733 | 6851324 | -2.12 |
| Retratricopeptide repeat domain 5 | *Ttc5* | 219022 | 6824550 | -2.12 |
| Basic helix-loop-helix family, member e40 | *Bhlhe40* | 20893 | 6948913 | -2.10 |
| Plasminogen activator, tissue | *Plat* | 18791 | 6974639 | -2.10 |
| Regulator of G-protein signaling 4 | *Rgs4* | 19736 | 6763991 | -2.09 |
| Gamma-aminobutyric acid (GABA) A receptor, subunit alpha 3 | *Gabra3* | 14396 | 7017520 | -2.09 |
| Glycine amidinotransferase (L-arginine:glycineamidinotransferase) | *Gatm* | 67092 | 6890448 | -2.08 |
| N-acylethanolamine acid amidase | *Naaa* | 67111 | 6939985 | -2.08 |
| Glycoprotein (transmembrane) nmb | *Gpnmb* | 93695 | 6946055 | -2.08 |
| Mitochondrial ribosomal protein S17 | *Mrps17* | 66258 | 6934631 | -2.07 |
| Mast cell expressed membrane protein 1 | *Mcemp1* | 69189 | 6973693 | -2.07 |
| RIKEN cDNA 5830428M24 gene | *5830428M24Rik* | 76062 | 6801480 | -2.07 |
| Glucagon-like peptide 1 receptor | *Glp1r* | 14652 | 6849762 | -2.06 |
| RIKEN cDNA A330076H08 gene | *A330076H08Rik* | 320026 | 6967799 | -2.06 |
| High mobility group AT-hook 2, pseudogene 1 | *Hmga2-ps1* | 15365 | 6764405 | -2.04 |
| Cytoplasmic polyadenylation element binding protein 1 | *Cpeb1* | 12877 | 6968828 | -2.04 |
| Cholinergic receptor, muscarinic 3, cardiac | *Chrm3* | 12671 | 6804725 | -2.03 |
| Macrophage galactose N-acetyl-galactosamine specific lectin 2 | *Mgl2* | 216864 | 6782102 | -2.03 |
| RIKEN cDNA 9930104M19 gene | *9930104M19Rik* | 320788 | 6812907 | -2.03 |
| Sodium channel, nonvoltage-gated 1 alpha | *Scnn1a* | 20276 | 6949860 | -2.02 |
| Lymphocyte antigen 75 | *Ly75* | 17076 | 6887081 | -2.02 |
| Secreted Ly6/Plaur domain containing 1 | *Slurp1* | 57277 | 6836694 | -2.02 |
| RIKEN cDNA 3110035G12 gene | *3110035G12Rik* | 73169 | 6953902 | -2.01 |
| Caspase 12 | *Casp12* | 12364 | 6986651 | -2.00 |
| Functional intergenic repeating RNA element | *Firre* | 103012 | 7016782 | -2.00 |

**Table S7**. Transcripts showing consistent pattern of expression lung transcripts in JF1/MsJ (JF1) compared to C3H/HeJ (C3H) mice across E18/ P28 stages with cut off for fold change ≥2 fold.in both stages [increased transcripts=16; decreased transcripts=28]; false discovery rate <10%

| **Gene Name** | **Gene symbol** | **Entrez ID** | **Probeset** | **JF1/C3H** | |
| --- | --- | --- | --- | --- | --- |
|  |  |  |  | **E18** | **P28** |
| **Increased transcripts** |  |  |  |  |  |
| Glutaredoxin 3 | *Glrx3* | 30926 | 6923313 | 12.22 | 6.08 |
| RIKEN cDNA 6330403K07 gene | *6330403K07Rik* | 103712 | 6789483 | 2.32 | 7.15 |
| RIKEN cDNA 2610507I01 gene | *2610507I01Rik* | 72203 | 6788659 | 2.60 | 5.35 |
| Olfactory receptor 170 | *Olfr170* | 258959 | 6844403 | 2.47 | 4.67 |
| Predicted gene 14406 | *Gm14406* | 100504328 | 6894013 | 4.58 | 3.07 |
| RIKEN cDNA G730007D18 gene | *G730007D18Rik* | 100038502 | 7006004 | 3.66 | 2.87 |
| Holliday junction recognition protein | *Hjurp* | 381280 | 6760490 | 3.58 | 2.09 |
| Synaptonemal complex central element protein 1 | *Syce1* | 74075 | 6972143 | 3.00 | 2.06 |
| Predicted gene 14403 | *Gm14403* | 433520 | 6883977 | 2.98 | 2.79 |
| RIKEN cDNA 2610028J07 gene | *2610028J07Rik* | 71813 | 6859453 | 2.94 | 2.74 |
| Olfactory receptor 1061 | *Olfr1061* | 259022 | 6888451 | 2.51 | 2.81 |
| Microspherule protein 1 | *Mcrs1* | 51812 | 6918337 | 2.62 | 2.03 |
| Lipopolysaccharide binding protein | *Lbp* | 16803 | 6882730 | 2.36 | 2.28 |
| Uncharacterized protein C130090J04 | *C130090J04* | 328049 | 6860959 | 2.36 | 2.12 |
| Aldo-keto reductase family 1, member E1 | *Akr1e1* | 56043 | 6810717 | 2.16 | 2.03 |
| Solute carrier family 22 (organic anion transporter), member 19 | *Slc22a19* | 207151 | 6871332 | 2.03 | 2.01 |
| **Decreased Transcripts** |  |  |  |  |  |
| Hemolytic complement | *Hc* | 15139 | 6886022 | -8.61 | -32.16 |
| Kallikrein 1-related peptidase b21 | *Klk1b21* | 16616 | 6960239 | -8.25 | -19.87 |
| RIKEN cDNA A730017L22 gene | *A730017L22Rik* | 613258 | 6890967 | -13.13 | -15.62 |
| Chemokine (C-X-C motif) ligand 15 | *Cxcl15* | 20309 | 6932372 | -14.60 | -2.25 |
| Ring finger protein 19A pseudogene | *Gm7444* | 665005 | 6989287 | -12.33 | -3.13 |
| Zinc finger, X-linked, duplicated B | *Zxdb* | 668166 | 7021109 | -6.18 | -7.68 |
| G protein-coupled receptor 137B | *Gpr137b* | 83924 | 7003081 | -5.39 | -5.38 |
| Sodium channel, voltage-gated, type III, alpha | *Scn3a* | 20269 | 6887324 | -2.08 | -5.31 |
| RIKEN cDNA B930063I24 gene | *B930063I24Rik* | 319330 | 6756745 | -5.06 | -4.33 |
| Transmembrane protein 181A | *Tmem181a* | 77106 | 6812918 | -4.26 | -4.08 |
| RIKEN cDNA 1300014J16 gene | *1300014J16Rik* | 74160 | 6796370 | -4.02 | -2.88 |
| Surfactant associated 2 | *Sfta2* | 433102 | 6850183 | -4.02 | -2.19 |
| RIKEN cDNA D230046O15 gene | *D230046O15Rik* | 106824 | 6856766 | -3.00 | -3.63 |
| Nuclear paraspeckle assembly transcript 1 (non-protein coding) | *Neat1* | 66961 | 6867774 | -3.54 | -3.45 |
| Interferon-induced protein 44 | *Ifi44* | 99899 | 6910592 | -2.10 | -2.86 |
| Expressed sequence AL118220 | *AL118220* | 404583 | 6815681 | -2.83 | -2.04 |
| Aminolevulinate, delta-, dehydratase | *Alad* | 17025 | 6922241 | -2.82 | -2.54 |
| Megalencephalicleukoencephalopathy with subcortical cysts 1 homolog (human) | *Mlc1* | 170790 | 6837773 | -2.24 | -2.78 |
| Sterile alpha motif domain containing 9-like | *Samd9l* | 209086 | 6951281 | -2.08 | -2.74 |
| Transmembrane protein 181A | *Tmem181a* | 77106 | 6848520 | -2.13 | -2.60 |
| RIKEN cDNA A330076H08 gene | *A330076H08Rik* | 320026 | 6967799 | -2.35 | -2.53 |
| NME/NM23 family member 7 | *Nme7* | 171567 | 6754701 | -2.13 | -2.49 |
| Cytochrome P450, family 2, subfamily j, polypeptide 6 | *Cyp2j6* | 13110 | 6923520 | -2.29 | -2.27 |
| Solute carrier family 39 (metal ion transporter), member 8 | *Slc39a8* | 67547 | 6901634 | -2.07 | -2.43 |
| Glucagon-like peptide 1 receptor | *Glp1r* | 14652 | 6849762 | -2.02 | -2.33 |
| Predicted gene 10563 | *Gm10563* | 100038426 | 6927270 | -2.11 | -2.25 |
| Alcohol dehydrogenase 1 (class I) | *Adh1* | 11522 | 6901737 | -2.24 | -2.00 |
| Protein phosphatase 1, regulatory (inhibitor) subunit 3A | *Ppp1r3a* | 140491 | 6951783 | -2.19 | -2.10 |

**Table S8**. Transcripts showing consistent pattern of expression lung transcripts in JF1/MsJ (JF1) compared to C3H/HeJ (C3H) mice across P28/ P70 stages with cut off for fold change 2.0 fold.in both stages [increased transcripts=32; decreased transcripts=70]; false discovery rate <10%

| **Gene Name** | **Gene symbol** | **Entrez ID** | **Probeset** | **JF1/C3H** | |
| --- | --- | --- | --- | --- | --- |
|  |  |  |  | **P28** | **P70** |
| **Increased transcripts** |  |  |  |  |  |
| Serine peptidase inhibitor, Kazal type 5 | *Spink5* | 72432 | 6860513 | 12.79 | 9.13 |
| WAP four-disulfide core domain 10 | *Wfdc10* | 629756 | 6883109 | 9.45 | 3.48 |
| Olfactory receptor 170 | *Olfr170* | 258959 | 6844409 | 7.88 | 5.47 |
| RIKEN cDNA 6330403K07 gene | *6330403K07Rik* | 103712 | 6789483 | 7.15 | 6.56 |
| Olfactory receptor 170 | *Olfr170* | 258959 | 6844407 | 6.81 | 4.24 |
| Follistatin | *Fst* | 14313 | 6816226 | 6.41 | 4.57 |
| Glutaredoxin 3 | *Glrx3* | 30926 | 6923313 | 6.08 | 3.27 |
| Wnt inhibitory factor 1 | *Wif1* | 24117 | 6771207 | 6.02 | 5.42 |
| RIKEN cDNA B430010I23 gene | *B430010I23Rik* | 78849 | 6975675 | 5.95 | 5.82 |
| Ankyrin repeat domain 63 | *Ankrd63* | 383787 | 6890143 | 5.93 | 5.01 |
| Arylsulfatasei | *Arsi* | 545260 | 6861349 | 5.71 | 4.17 |
| RIKEN cDNA 2610507I01 gene | *2610507I01Rik* | 72203 | 6788659 | 5.35 | 3.74 |
| Olfactory receptor 170 | *Olfr170* | 258959 | 6844403 | 4.67 | 3.43 |
| RIKEN cDNA 1700093K21 gene | *1700093K21Rik* | 67358 | 6786660 | 3.12 | 2.62 |
| RIKEN cDNA G730007D18 gene | *G730007D18Rik* | 100038502 | 7006004 | 2.87 | 2.41 |
| RIKEN cDNA E230023K05 gene | *E230023K05Rik* | 319435 | 6828485 | 2.82 | 2.63 |
| Olfactory receptor 1061 | *Olfr1061* | 259022 | 6888451 | 2.81 | 3.77 |
| Radial spoke head 1 homolog (Chlamydomonas) | *Rsph1* | 22092 | 6854792 | 2.81 | 2.63 |
| Predicted gene 14403 | *Gm14403* | 433520 | 6883977 | 2.79 | 2.68 |
| RIKEN cDNA 2610028J07 gene | *2610028J07Rik* | 71813 | 6859453 | 2.74 | 2.82 |
| Ventricular zone expressed PH domain homolog 1 (zebrafish) | *Veph1* | 72789 | 6905660 | 2.04 | 2.49 |
| Predicted gene 2115 | *Gm2115* | 100039239 | 6962238 | 2.47 | 2.05 |
| Microspherule protein 1 | *Mcrs1* | 51812 | 6918337 | 2.03 | 2.30 |
| Rho GTPase activating protein 20 | *Arhgap20* | 244867 | 6989086 | 2.29 | 2.21 |
| Acyl-CoA synthetase medium-chain family member 1 | *Acsm1* | 117147 | 6963886 | 2.26 | 2.28 |
| Histocompatibility 2, D region locus 1 | *H2-D1* | 14964 | 6850135 | 2.21 | 2.11 |
| Uncharacterized protein C130090J04 | *C130090J04* | 328049 | 6860959 | 2.12 | 2.20 |
| Holliday junction recognition protein | *Hjurp* | 381280 | 6760490 | 2.09 | 2.20 |
| Synaptonemal complex central element protein 1 | *Syce1* | 74075 | 6972143 | 2.06 | 2.13 |
| Solute carrier family 1 (glutamate/neutral amino acid transporter), member 4 | *Slc1a4* | 55963 | 6786473 | 2.01 | 2.09 |
| Cancer susceptibility candidate 4 | *Casc4* | 319996 | 6880658 | 2.08 | 2.00 |
| Doublecortin domain containing 2b | *Dcdc2b* | 100504491 | 6925585 | 2.04 | 2.04 |
| **Decreased Transcripts** |  |  |  |  |  |
| Hemolytic complement | *Hc* | 15139 | 6886022 | -32.16 | -27.88 |
| Kallikrein 1-related peptidase b21 | *Klk1b21* | 16616 | 6960239 | -19.87 | -11.92 |
| Immunoglobulin kappa chain variable 4-91 | *Igkv4-91* | 434033 | 6954474 | -4.38 | -16.44 |
| Chitinase 3-like 3 | *Chi3l3* | 12655 | 6907945 | -15.62 | -15.93 |
| RIKEN cDNA A730017L22 gene | *A730017L22Rik* | 613258 | 6890967 | -15.62 | -14.60 |
| Uncharacterized LOC624295 | *LOC624295* | 624295 | 6792817 | -10.82 | -7.19 |
| Zinc finger, X-linked, duplicated B | *Zxdb* | 668166 | 7021109 | -7.68 | -7.23 |
| Predicted gene 4956 | *Gm4956* | 241041 | 6757322 | -7.19 | -4.40 |
| Sciellin | *Scel* | 64929 | 6821514 | -3.35 | -7.16 |
| RIKEN cDNA B930063I24 gene | *B930063I24Rik* | 319330 | 6756745 | -4.33 | -6.19 |
| Sulfotransferase family 1D, member 1 | *Sult1d1* | 53315 | 6939723 | -3.52 | -6.05 |
| BAI1-associated protein 2-like 1 | *Baiap2l1* | 66898 | 6942960 | -4.53 | -5.92 |
| Cryptochrome 2 (photolyase-like) | *Cry2* | 12953 | 6888835 | -2.15 | -5.41 |
| G protein-coupled receptor 137B | *Gpr137b* | 83924 | 7003081 | -5.38 | -3.06 |
| ATP-binding cassette, sub-family A (ABC1), member 8a | *Abca8a* | 217258 | 6792114 | -3.16 | -5.38 |
| Sodium channel, voltage-gated, type III, alpha | *Scn3a* | 20269 | 6887324 | -5.31 | -4.49 |
| C-type lectin domain family 4, member n | *Clec4n* | 56620 | 6949744 | -4.88 | -5.15 |
| Histocompatibility 2, class II antigen E alpha, pseudogene | *H2-Ea-ps* | 100504404 | 6855022 | -4.29 | -4.35 |
| RIKEN cDNA 5730424H11 gene | *5730424H11Rik* | 100038701 | 6832562 | -3.06 | -4.29 |
| Predicted gene 10673 | *Gm10673* | 100038559 | 6907955 | -4.22 | -2.33 |
| Transmembrane protein 181A | *Tmem181a* | 77106 | 6812918 | -4.08 | -3.40 |
| Chemokine (C-C motif) ligand 6 | *Ccl6* | 20305 | 6790290 | -3.20 | -3.92 |
| Leukocyte immunoglobulin-like receptor, subfamily B, member 4 | *Lilrb4* | 14728 | 6767782 | -3.80 | -3.64 |
| Cathepsin S | *Ctss* | 13040 | 6899683 | -3.07 | -3.73 |
| ATPase, H+ transporting, lysosomal V0 subunit D2 | *Atp6v0d2* | 242341 | 6920056 | -3.27 | -3.64 |
| RIKEN cDNA D230046O15 gene | *D230046O15Rik* | 106824 | 6856766 | -3.63 | -2.54 |
| Leukocyte immunoglobulin-like receptor, subfamily A (with TM domain), member 5 | *Lilra5* | 232801 | 6972927 | -2.57 | -3.63 |
| Stathmin-like 2 | *Stmn2* | 20257 | 6895589 | -3.62 | -3.37 |
| matrix metallopeptidase 3 | *Mmp3* | 17392 | 6986725 | -2.45 | -3.62 |
| Guanylate binding protein 1 | *Gbp1* | 14468 | 6901952 | -3.11 | -3.47 |
| Nuclear paraspeckle assembly transcript 1 (non-protein coding) | *Neat1* | 66961 | 6867774 | -3.45 | -2.70 |
| Chitinase, acidic | *Chia* | 81600 | 6900239 | -3.33 | -2.26 |
| Histocompatibility 2, Q region locus 4 | *H2-Q4* | 15015 | 6850155 | -3.19 | -2.86 |
| Megalencephalicleukoencephalopathy with subcortical cysts 1 homolog (human) | *Mlc1* | 170790 | 6837773 | -2.78 | -3.13 |
| Protein phosphatase 1, regulatory (inhibitor) subunit 3A | *Ppp1r3a* | 140491 | 6951783 | -2.10 | -3.06 |
| Sterile alpha motif domain containing 9-like | *Samd9l* | 209086 | 6951281 | -2.74 | -3.05 |
| Interferon-induced protein 44 | *Ifi44* | 99899 | 6910592 | -2.86 | -2.19 |
| Myxovirus (influenza virus) resistance 2 | *Mx2* | 17858 | 6843550 | -2.79 | -2.26 |
| Macrophage expressed gene 1 | *Mpeg1* | 17476 | 6868171 | -2.74 | -2.71 |
| NME/NM23 family member 7 | *Nme7* | 171567 | 6754701 | -2.49 | -2.70 |
| Coiled-coil domain containing 190 | *Ccdc190* | 78465 | 6755059 | -2.41 | -2.69 |
| Aminolevulinate, delta-, dehydratase | *Alad* | 17025 | 6922241 | -2.54 | -2.67 |
| cDNA sequence BC064078 | *BC064078* | 408064 | 6950112 | -2.67 | -2.20 |
| Fatty acid binding protein 1, liver | *Fabp1* | 14080 | 6946900 | -2.14 | -2.67 |
| Integrin alpha X | *Itgax* | 16411 | 6964382 | -2.35 | -2.66 |
| Ankyrin repeat domain 1 (cardiac muscle) | *Ankrd1* | 107765 | 6872834 | -2.28 | -2.66 |
| CD68 antigen | *Cd68* | 12514 | 6789325 | -2.47 | -2.63 |
| Transmembrane protein 181A | *Tmem181a* | 77106 | 6848520 | -2.60 | -2.40 |
| Macrophage galactose N-acetyl-galactosamine specific lectin 2 | *Mgl2* | 216864 | 6782102 | -2.56 | -2.03 |
| Transmembrane protein 2 | *Tmem2* | 83921 | 6868650 | -2.21 | -2.55 |
| CD209a antigen | *Cd209a* | 170786 | 6980091 | -2.54 | -2.30 |
| RIKEN cDNA A330076H08 gene | *A330076H08Rik* | 320026 | 6967799 | -2.53 | -2.06 |
| ST6 (alpha-N-acetyl-neuraminyl-2,3-beta-galactosyl-1,3)-N-acetylgalactosaminide alpha-2,6-sialyltransferase 2 | *St6galnac2* | 20446 | 6792544 | -2.47 | -2.52 |
| Solute carrier family 39 (metal ion transporter), member 8 | *Slc39a8* | 67547 | 6901634 | -2.43 | -2.51 |
| C-type lectin domain family 9, member a | *Clec9a* | 232414 | 6950148 | -2.26 | -2.48 |
| Bone morphogenetic protein 3 | *Bmp3* | 319746 | 6932719 | -2.43 | -2.33 |
| High mobility group AT-hook 2, pseudogene 1 | *Hmga2-ps1* | 15365 | 6764405 | -2.42 | -2.04 |
| Cytochrome P450, family 2, subfamily j, polypeptide 6 | *Cyp2j6* | 13110 | 6923520 | -2.27 | -2.39 |
| Glucagon-like peptide 1 receptor | *Glp1r* | 14652 | 6849762 | -2.33 | -2.06 |
| CD74 antigen (invariant polypeptide of major histocompatibility complex, class II antigen-associated) | *Cd74* | 16149 | 6861341 | -2.32 | -2.20 |
| Protein kinase, cGMP-dependent, type II | *Prkg2* | 19092 | 6940300 | -2.31 | -2.29 |
| Cathepsin K | *Ctsk* | 13038 | 6899682 | -2.31 | -2.28 |
| RIKEN cDNA A530099J19 gene | *A530099J19Rik* | 319293 | 6811368 | -2.02 | -2.24 |
| Histocompatibility 2, class II antigen E beta | *H2-Eb1* | 14969 | 6849968 | -2.23 | -2.21 |
| Surfactant associated 2 | *Sfta2* | 433102 | 6850183 | -2.19 | -2.23 |
| RIKEN cDNA 5830428M24 gene | *5830428M24Rik* | 76062 | 6801480 | -2.18 | -2.07 |
| KLRAQ motif containing 1 | *Klraq1* | 73825 | 6852929 | -2.04 | -2.18 |
| Placenta expressed transcript 1 | *Plet1* | 76509 | 6989010 | -2.09 | -2.16 |
| Glycine amidinotransferase (L-arginine:glycineamidinotransferase) | *Gatm* | 67092 | 6890448 | -2.08 | -2.08 |
| N-acylethanolamine acid amidase | *Naaa* | 67111 | 6939985 | -2.08 | -2.08 |

**Supplementary figure legends:**

**Supplementary Figure S1:**Representative lung sections showing smaller alveoli in JF1/Msf (JF1) mice compared to C3H/HeJ (C3H) in both males and females (identical magnification: 20X).**B.** Mean chord length (Lcm) of the alveolar airspace is smaller in JF1 mice compared to C3H among both sexes.**C.**Transmission electron microscopic (TEM) quantification of lung tissues by design-based stereology demonstrates that the major cellular compartments were not significantly different between C3H and JF1 mice. **D.** Representative TEM photographs of cross-sections through alveolar entrance rings of alveolar septum, which exhibit fibrocytes (**Fib),** collagen (**Col**) and elastin (**Ela**) fibers in C3H and JF1 mouse lungs.Alveolar septa shows higher elastin-to-collagen ratio in JF1-mice compared to C3H.Data are represented as mean ± standard deviation; n=5; different letters within the figures indicate significant difference of means at p<0.05 (ANOVA, TUKEYs posthoc test)

**Supplementary Figure 1:**


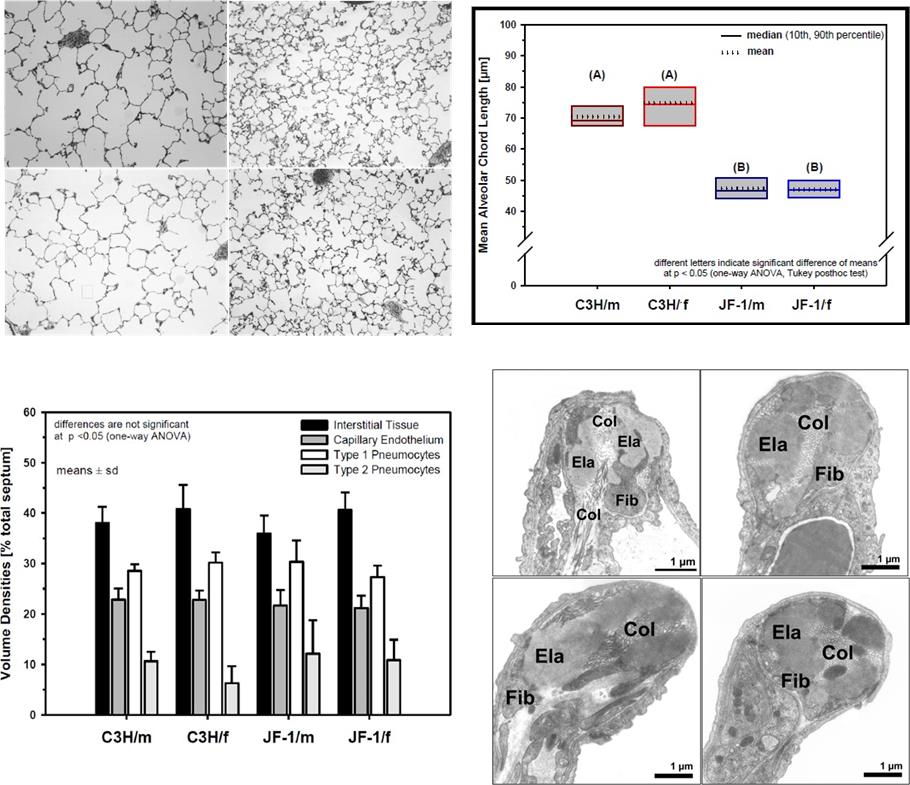


**D**

**C**

**B**

**A**
